# Single‐Cell and Spatial Transcriptomics Reveals a Stereoscopic Response of Rice Leaf Cells to *Magnaporthe oryzae* Infection

**DOI:** 10.1002/advs.202416846

**Published:** 2025-03-24

**Authors:** Wei Wang, Xianyu Zhang, Yong Zhang, Zhe Zhang, Chang Yang, Wen Cao, Yuqin Liang, Qinzheng Zhou, Qian Hu, Yimai Zhang, Yu Wang, Yingying Xing, Wenfeng Qian, Nan Yao, Ning Xu, Jun Liu

**Affiliations:** ^1^ State Key Laboratory of Agricultural and Forestry Biosecurity MOA Key Lab of Pest Monitoring and Green Management College of Plant Protection China Agricultural University Beijing 100193 China; ^2^ School of Computer Science Northwestern Polytechnical University Xi'an 710129 China; ^3^ MGI Tech QingDao 266426 China; ^4^ State Key Laboratory of Biocontrol Guangdong Provincial Key Laboratory of Plant Resources School of Life Sciences Sun Yat‐sen University Guangzhou 510275 China; ^5^ State Key Laboratory of Plant Genomics Institute of Genetics and Developmental Biology Innovation Academy for Seed Design Chinese Academy of Sciences Beijing 100101 China

**Keywords:** Magnaporthe oryzae‐rice interaction, longitudinal immunity, single‐cell transcriptomics, spatial transcriptomics, vascular immunity

## Abstract

Infection by the fungal pathogen *Magnaporthe oryzae* elicits dynamic responses in rice. Utilizing an integrated approach of single‐cell and spatial transcriptomics, a 3D response is uncovered within rice leaf cells to *M. oryzae* infection. A comprehensive rice leaf atlas is constructed from 236 708 single‐cell transcriptomes, revealing heightened expression of immune receptors, namely Pattern Recognition Receptors (PRRs) and Nucleotide‐binding site and leucine‐rich repeat (NLRs) proteins, within vascular tissues. Diterpene phytoalexins biosynthesis genes are dramatically upregulated in procambium cells, leading to an accumulation of these phytoalexins within vascular bundles. Consistent with these findings, microscopic observations confirmed that *M. oryzae* is prone to target leaf veins for invasion, yet is unable to colonize further within vascular tissues. Following fungal infection, basal defenses are extensively activated in rice cells, as inferred from trajectory analyses. The spatial transcriptomics reveals that rice leaf tissues toward leaf tips display stronger immunity. Characterization of the polarity gene *OsHKT9* suggests that potassium transport plays a critical role in resisting *M. oryzae* infection by expression along the longitudinal axis, where the immunity is stronger toward leaf tip. This work uncovers that there is a cell‐specific and multi‐dimensional (local and longitudinal) immune response to a fungal pathogen infection.

## Graphical abstract

By employing a combination of single cell and spatial transcriptomic sequencing, this study presents a stereoscopic response of rice leaf to *Magnaporthe oryzae* infection. The vascular tissues mount defenses by producing phytoalexins. The immune strength is stronger toward the rice leaf tip than that of the leaf base.

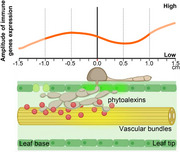

## 1 Introduction

Plants are constantly exposed to various microorganisms, many of which are pathogenic. To cope with these pathogen challenges, resistant plants have developed an elegant immune system that protects them from devastating infection. This immune system comprises plasma membrane‐located receptors and intracellular receptors, namely the pattern recognition receptors (PRRs) and the nucleotide‐binding site and leucine‐rich repeat (NLR) proteins. Activation of these receptors mediates immune responses that can prevent pathogen invasion. PRRs and NLRs are widely distributed in the plant kingdom.^[^ ^1^ ^]^ For example, *Arabidopsis* plants possess ≈640 PRRs and 160 NLRs,^[^ ^2^, ^3^ ^]^ while rice plants harbor ≈1100 PRRs and over 400 NLRs.^[^ ^4^, ^5^ ^]^ PRRs recognize conserved features of pathogens to initiate immunity, forming a first line of defense against pathogens. However, many pathogens have evolved virulence effectors to evade PRR‐mediated immunity. Nevertheless, resistant plants have evolved cognate NLRs to recognize these effectors and trigger a stronger immune response, known as the hypersensitive response.

The dynamic process of immune receptor‐mediated signal transduction in plants exhibits both temporal and spatial intricacies. Early immune responses encompass Ca^2+^ influx, reactive oxygen species (ROS) burst, and MAPK activation, while subsequent responses involve callose deposition, ethylene production, defense gene expression, and inhibition of plant growth.^[^ ^6^ ^]^ These responses typify the outcomes of PRRs activation. NLRs‐triggered immunity often culminates in localized cell death and the induction of systemic acquired resistance (SAR), thereby fortifying distant tissues against pathogen intrusion. Furthermore, plants synthesize antimicrobial compounds, such as phytoanticipins and phytoalexins, to bolster their defense mechanisms.

Pathogens exhibit varied lifestyles, including biotrophs, hemibiotrophs, and necrotrophs. These different modes of life often entail diverse interactions with plant cells during infection, eliciting distinct responses. Biotrophic pathogens proliferate in living tissues without causing severe host tissue destruction. In contrast, necrotrophic pathogens destroy host tissue and thrive on dead tissue. Hemibiotrophic pathogens initially coexist with the host but eventually kill it. Furthermore, many pathogens undergo morphological changes and allocate nutrients among cells during infection, particularly fungal and oomycete pathogens. Some of these pathogens produce numerous spores, which, upon landing on plant tissue, swiftly germinate and form appressoria or similar structures to penetrate the host epidermis. In the case of hemibiotrophic fungal pathogens, they subsequently develop invasive hyphae (IH) and spread within plant tissues. However, bacterial pathogens do not undergo notable changes in appearance but display a preference for colonizing specific host niches, such as leaf veins, crevices, and trichomes, leading to uneven infection patterns within leaves. This nonuniform infection results in a cell‐specific temporal and spatial immune response in plants.^[^ ^7^ ^]^

Plant cell heterogeneity may contribute to differential immune responses among cells. In the compatible *Blumeria graminis f*. sp. *hordei*‐barley interaction, it has been observed that certain barley leaf epidermal cells become infected while others resist penetration.^[^ ^8^ ^]^ This distinct resistance capacity is also evident across different organs. For example, numerous rice NLR genes induce resistance against leaf blast at the seedling stage, but only a limited number confers resistance to panicle blast.^[^ ^9^ ^]^ Through transcriptome sequencing of rice leaf segments, Li et al. revealed uneven gene expression along the longitudinal axis.^[^ ^10^ ^]^ Bulk RNA sequencing may overlook the expression of genes with low profiles that could be highly expressed in specific cells. To accurately capture the transcripts of cell‐specific responsive genes, various techniques have been developed, including laser‐microdissection, cell sorting and enrichment, in‐situ hybridization, and single‐cell sequencing. Among these, single‐cell RNA sequencing (scRNA‐seq) offers significant advantages due to its high throughput capacity for identifying various cell populations. Spatiotemporal RNA sequencing has also gained prominence for investigating gene expression at a spatial level, surpassing traditional in‐situ hybridization or related techniques. These methodologies enable the exploration of dynamic temporal and spatial gene expression patterns in plant cells.

In this study, we utilized single‐cell nucleus RNA sequencing (snRNA‐seq) and spatial‐temporal transcriptome sequencing (stRNA‐seq) techniques to elucidate the responses of plant cells to rice blast infection caused by *M. oryzae*. These techniques were employed to circumvent potential artifacts affecting transcript abundance introduced during experimentation.^[^ ^11^, ^12^ ^]^ *M. oryzae* is a representative hemibiotrophic fungal pathogen, with the infection process in rice suggested to involve a significant infection style change. It is postulated that the early infection is at biotrophic stage while the later infection is at necrotrophic stage. Despite the complexity of *M. oryzae* infection, it serves as an ideal model for dissecting cell‐specific responses in monocot‐hemibiotrophic pathogen interactions. Employing snRNA‐seq and stRNA‐seq techniques, we examined a compatible interaction involving the fungal strain Guy11 and rice variety *Nipponbare*. Our findings indicate that PRRs and NLRs actively participated in rice immunity at 48 hpi in mestome sheath cells, potentially defining a compatible interaction. However, downstream signaling activated by PRRs was observed in all leaf tissues post‐infection. Notably, phytoalexins were primarily produced in procambium cells and immune receptors were highly expressed in vascular tissues, likely serving as the crucial defense mechanisms against pathogen invasion in the vascular region. Furthermore, stRNA‐seq results revealed a polarized immune response in leaf tissues, with immunity favoring the leaf tip along the longitudinal axis, potentially influencing the final lesion shape. Thus, we present a comprehensive understanding of rice leaf cell responses to *M. oryzae* infection.

## 2 Results

### 2.1 Cell Transcriptome Atlas of *M. oryzae*‐Infected Rice Leaves

**Figure 1:**
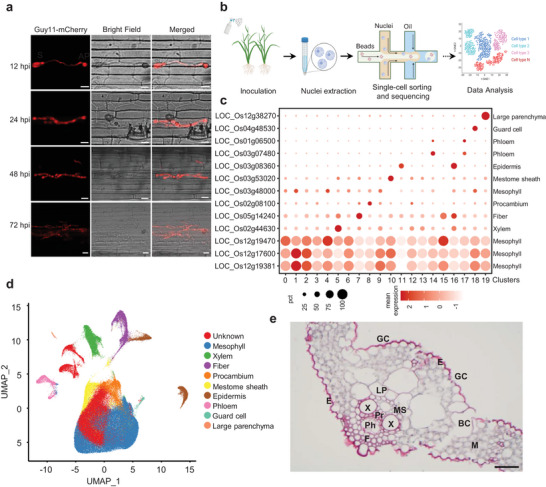
Transcriptome atlas of rice leaf cells infected with *M. oryzae*. a) Micrographs of rice leaf sheath inoculated with *M. oryzae*. *M. oryzae* strain Guy11 expressing mCherry red fluorescence was used to observe the infection. Images were taken at 12, 24, 48, and 72 h post‐inoculation (hpi). Scale bars, 10 µm. b) Schematic diagram of the single nucleus transcriptomics library preparation, sequencing, and analysis. c) Dot plot of the mean expression levels and percent of cells expressing cell‐specific marker genes in different clusters in the integrated snRNA‐seq data. The data were integrated from all sequenced cells. The cell‐specific markers are from Wang et al.^[^ ^16^ ^]^ d) A transcriptome atlas consisting of 236,708 cells. Each cell was represented by a dot on a plot visualized by uniform manifold approximation and projection (UMAP). According to Graph‐based unsupervised clustering, the cells were divided into 20 clusters using graph‐based, unsupervised clustering. The clusters were assigned to 9 cell types and the unknown cell types. e) The paraffin sectioning image of the cross dissection of rice leaf vein. A two‐week‐old rice leaf was used for the cross‐dissection. The cell‐specific tissues were indicated with letters. The tissues were stained with safranine, and the photo was taken using a light microscope. M, Mesophyll; X, Xylem; F, Fiber; Pr, Procambium; MS, Mestome sheath; E, Epidermis; GC, Guard cell; Ph, Phloem; LP, Large parenchyma; BC, Bulliform cell; GC, Guard cell. Scale bars, 20 µm.

In order to investigate the single‐cell transcriptome of rice leaves during *M. oryzae* infection, we initiated our study by examining the infection process of the pathogen. Two‐week‐old rice seedlings were subjected to spray inoculation with conidial suspensions of the *M. oryzae* strain Guy11 expressing the red fluorescent protein mCherry. Using confocal microscopy, we observed that at 12 h post‐inoculation (hpi), the spore formed a germ tube and an appressorium on the leaf surface. Subsequently, between 24 and 48 hpi, the fungal hyphae predominantly proliferated within the initially infected cells and began to invade neighboring cells. By 72 hpi, the fungal hyphae extended into the third neighboring cell (Figure **1**a).

To examine the early transcriptome at the single‐cell level, we sampled the infected leaves at 0, 12, 24, and 48 hpi for single‐nucleus RNA sequencing (snRNA‐seq). The experimental schematic for snRNA‐seq is illustrated in Figure 1b. We successfully obtained 236 708 high‐quality nuclei in total, with an average of 1307 detected genes per cell. Additionally, an average of 2724 unique molecular identifiers (UMIs) were captured for each cell (Supplementary Figure S1a and Table S1a, Supporting Information).^[^ ^13^ ^]^ These findings affirm the satisfactory quality of the snRNA‐seq data for subsequent analyses.

Leveraging the snRNA‐seq data, we utilized the Seurat R package^[^ ^14^ ^]^ to construct a single‐cell transcriptome atlas for infected rice leaves, wherein we identified 20 distinct cell clusters (Figure S1b, Supporting Information). By employing identified cell‐specific marker genes, AddModuleScore function in Seurat and GO enrichment analysis, which exhibited significant enrichment in the transcriptome,^[^ ^15^, ^16^, ^17^ ^]^ we further categorized the clusters into nine cell types and one unidentified cell group encompassing clusters 0, 3, 12, and 13 (Figure 1c,d; Figure S1c and Table S1b,c, Supporting Information). Notably, these nine cell types were consistently identified across all examined samples, underscoring the reliability of our data (Figure S2a, Supporting Information). Moreover, the actual cell types can be discerned based on cell morphologies observed through either light microscopy or scanning electron microscopy (SEM) (Figure 1e; Figure S2b, Supporting Information). Furthermore, all investigated samples contained the nine cell types within the 20 clusters. It is noteworthy that cluster “8,” designated as procambium cells, exhibited a high proportion of enrichment in *M. oryzae*‐infected samples at 48 hpi, which may be attributed to the stimulus of pathogen infection (Figure S2c, Supporting Information).

### 2.2 Spatiotemporal Cell‐Type‐Specific Expression of Immune‐Related Genes

**Figure 2:**
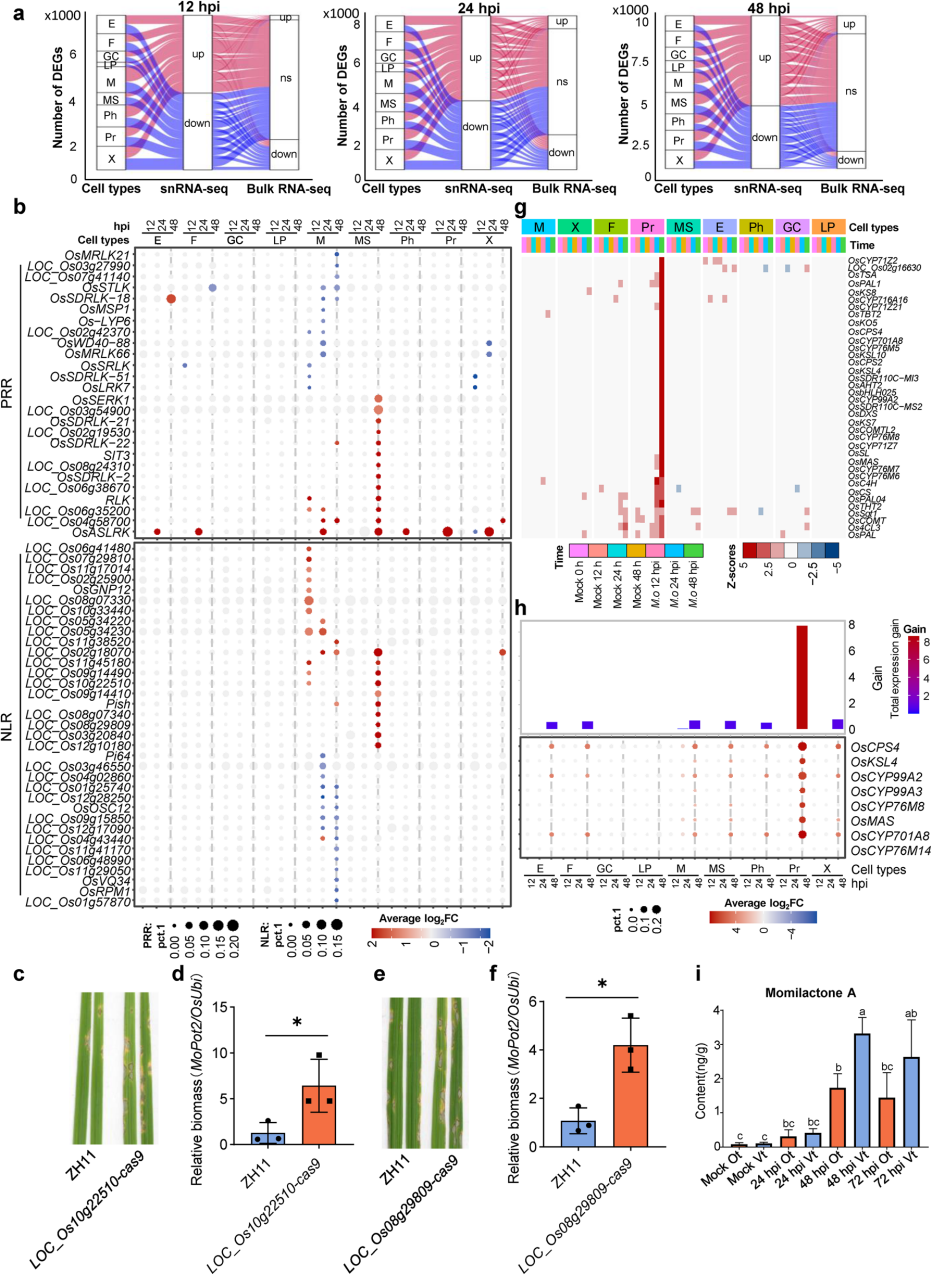
Spatiotemporal cell‐type‐specific expression of immune‐related genes. a) The differentially expressed genes (DEGs) identified by snRNA‐seq and bulk RNA‐seq. The DEGs identified by snRNA‐seq were masked in bulk transcriptome assays. Rice leaves were inoculated with *M. oryzae* spores and sampled at indicated time points. The bulk RNA‐seq data were retrieved from NCBI (PRJNA661210). DEGs from the snRNA‐seq data were detected by FindMarker in each annotated cell type using cutoffs of | average log_2_FC| > 1.0, *p* adj < 0.01, pct.1 > 0.2 for up‐regulated genes and pct.2 > 0.2 for down‐regulated genes. DEGs calling from the bulk RNA‐seq used cutoffs of |log_2_FC| > 1 and *p* adj < 0.01. M, Mesophyll; X, Xylem; F, Fiber; Pr, Procambium; MS, Mestome sheath; E, Epidermis; GC, Guard cell; Ph, Phloem; LP, Large parenchyma. NS, not significant. b) Dot plot of the average log_2_FC and percent of cell expression. Upper panel: Known PRR genes were expressed across different cell populations in the integrated snRNA‐seq data at 12, 24, and 48 hpi. Lower panel: Dot plot of NLR DEGs based on average log_2_FC value comparing with their control. |average log_2_FC| > 1.0, *p* adj < 0.01, pct.1 > 0.01 for up‐regulated genes and pct.2 > 0.01 for down‐regulated genes. M, Mesophyll; X, Xylem; F, Fiber; Pr, Procambium; MS, Mestome sheath; E, Epidermis; GC, Guard cell; Ph, Phloem; LP, Large parenchyma. c) *LOC_Os10g22510* mutant plants exhibited reduced disease resistance to *M. oryzae*. Disease symptoms were developed by spray inoculation with conidial suspensions at a concentration of 5 × 10^5^ spores mL^−1^. Images were taken at 5 dpi. d) The relative fungal biomass was determined by qPCR for the *M. oryzae Pot2* gene against rice *OsUbi* gene. Values are means ± SD. “*” represents *p* < 0.05 (Student's *t*‐test, *n* = 3 biological replicates). e) *LOC_Os08g29809* mutant plants exhibited reduced disease resistance to *M. oryzae*. Disease symptoms were developed by spray inoculation with conidial suspensions at a concentration of 5 × 10^5^ spores mL^−1^. Images were taken at 5 dpi. f) The relative fungal biomass was determined by qPCR for the *M. oryzae Pot2* gene against rice *OsUbi* gene. Values are means ± SD. “*” represents *p* < 0.05 (Student's *t*‐test, *n* = 3 biological replicates). g) Cell‐type‐specific expression of metabolic genes during *M. oryzae* infection. Heatmaps show mean expression levels of metabolic genes from rice cells in each annotated cell type based on colored Z‐scores. Others are same as in b). h) Expression of momilactone biosynthesis genes. Upper panel: total gain value of key momilactone biosynthesis gene expression. Lower panel: dot plot of average log_2_FC in different cell types at 12, 24, and 48 hpi. Total gain value = average log_2_FC × pct.1. Pct.1 means the percentage of cells where the gene is detected in the treatment group. i) Measurement of momilactone A in different rice leaf tissues. The content of momilactone A in different rice leaf tissues was measured by LC‐MS/MS after inoculated with *M. oryzae* at 24, 48, and 72 hpi. Ot, other tissues; Vt, vascular tissues. Letters indicate the significant difference between the expression levels at different sites by one‐way ANOVA at *p* < 0.05 (*n* = 3 biological repeats). Error bars represent means ± SD.

To thoroughly assess the quality and accuracy of snRNA‐seq data, we conducted a comparative analysis with a previously published bulk RNA‐seq dataset.^[^ ^18^ ^]^ We cross‐referenced the differentially expressed genes (DEGs) identified in distinct single cell types with those from bulk transcriptome data at 12, 24, and 48 hpi. About 75% of the DEGs were discernible by snRNA‐seq but not by bulk RNA sequencing (Figure **2**a; Table S2a, Supporting Information). This finding underscores the efficacy of snRNA‐seq in detecting “lower profile DEGs” at the single‐cell level, which are often elusive in bulk RNA‐seq analyses.

Pattern‐triggered immunity (PTI) mediated by PRRs is pivotal in early immune responses. Thus, we scrutinized the expression of PRRs^[^ ^19^, ^20^, ^21^ ^]^ in the identified cell populations within infected leaf samples. We detected the expression of 38 PRRs primarily in xylem, fiber, procambium, mestome sheath, and large parenchyma cells, while conspicuously absent in mesophyll, epidermis, guard cells, and phloem cells at 24 and 48 hpi (Figure S3a; Table S2b, Supporting Information). In order to reveal the actual expression levels of the genes, we also analyzed the PRR gene expression in the cell types, where we found that PRR expression levels were generally low across all cell types at 12 hpi, many DEGs induced only in mestome sheath cells at 48 hpi (Figure 2b; Table S2c, Supporting Information). On the other hand, NLRs,^[^ ^22^ ^]^ crucial for intracellular immune responses, displayed prominent expression primarily at 12 hpi, especially in mestome sheath and large parenchyma cells (Figure S3a; Table S2d, Supporting Information). Similar to PRRs, almost one‐third portion of NLR DEGs were induced in mestome sheath cells by 48 hpi (Figure 2b; Table S2e, Supporting Information). Together, these results imply that PRRs and NLRs were actively involved in rice immunity at 48 hpi in mestome sheath cells.

Among the up‐regulated PRRs, *OsASLRK*, a leucine‐rich repeat receptor‐like kinase (LRR‐RLK) encoding gene induced by various abiotic stresses,^[^ ^23^, ^24^ ^]^ was notably upregulated in almost all cell populations at 24 hpi (Figure 2b; Figure S3b, Supporting Information). To validate the role of *OsASLRK*, we conducted *M. oryzae* inoculation assays using *osaslrk* knockout plants. Surprisingly, *osaslrk*‐KO plants exhibited similar disease symptoms as the wild‐type HY (Hwayong background) plants (Figure S3c, Supporting Information). Among the induced cell‐type‐specific NLRs, *OsPish*, a well‐known resistance gene conferring resistance against *M. oryzae* isolates containing the effector AvrPish,^[^ ^25^ ^]^ was expressed across most cell types, with heightened induction in mestome sheath cells at 48 hpi (Figure 2b; Figure S3d, Supporting Information). To elucidate the function of induced NLRs, we selected several putative NLRs, *LOC_Os03g20840*, *LOC_Os08g29809*, and *LOC_Os10g22510*, exhibiting similar expression patterns as *OsPish* in mestome sheath cells at 48 hpi (Figure 2b; Figure S3e, Supporting Information), to investigate their recognition of *M. oryzae* effectors. By pathogen inoculation assays, we found that *NLRs* (*LOC_Os10g22510* and *LOC_Os08g29809*) knock‐out lines were more susceptible to *M. oryzae* in comparison with WT (Figure 2c–f). These findings suggest that certain induced NLRs, primarily expressed in mesophyll cells and mestome sheath, may recognize effectors from *M. oryzae*.

### 2.3 Spatiotemporal Cell‐Type‐Specific Expression of Phytoalexin Biosynthesis Genes

Given the high responsiveness of procambium cells to *M. oryzae* infection (Figure S2c, Supporting Information), we subsequently analyzed the transcriptome data to investigate the induction of other immune‐related signaling pathways in a cell‐type‐specific manner. Interestingly, we observed the specific activation of metabolism‐related pathways in procambium cells of infected samples at 24 and 48 hpi (Figure 2g; Table S3a, Supporting Information). Accordingly, the differentially expressed genes (DEGs) associated with metabolism were notably identified in procambium cells at 48 hpi (Figure S4a and Table S3b, Supporting Information). Annotation analysis of these genes revealed a significant enrichment in diterpenoid phytoalexin biosynthesis, with many of them being markedly induced by *M. oryzae* at 48 hpi (Figure S4a, Supporting Information). Diterpenoid phytoalexins, including momilactone A, oryzalexins, and phytocassanes, are well‐known antimicrobial compounds in rice (Table S3c, Supporting Information).^[^ ^26^, ^27^ ^]^ In particular, we identified eight key genes involved in momilactone biosynthesis (Figure S4b, Supporting Information), with seven of them showing substantial induction in procambium cells upon *M. oryzae* infection at 48 hpi (Figure 2h; Table S3d, Supporting Information). UMAP visualization indicated that four key genes, *OsCPS4*, *OsCYP99A2*, *OsCYP701A8*, and *OsCYP76M8*, were primarily expressed in procambium cells, although expression could also be detected in other cell types (Figure S4c, Supporting Information).^[^ ^28^ ^]^

Based on these findings, we postulate that rice vascular tissues play a pivotal role in momilactone A production during blast fungi infection. To validate this hypothesis, we quantified momilactone A levels using HPLC‐MS/MS in both vascular and non‐vascular tissues.^[^ ^29^ ^]^ At 24 hpi, we observed only marginal accumulation of momilactone A. However, by 48 hpi, there was a significant increase in momilactone A levels in vascular tissues as well as other parts of the leaf, with notably higher accumulation in vascular tissues compared to other tissues (Figure 2i). This outcome suggests that vascular tissue serves as the primary site for diterpenoid phytoalexin accumulation, thereby contributing to resistance against blast fungi infection in vascular tissues.

### 2.4 Veins are the Primary Targets of *M. oryzae*

**Figure 3:**
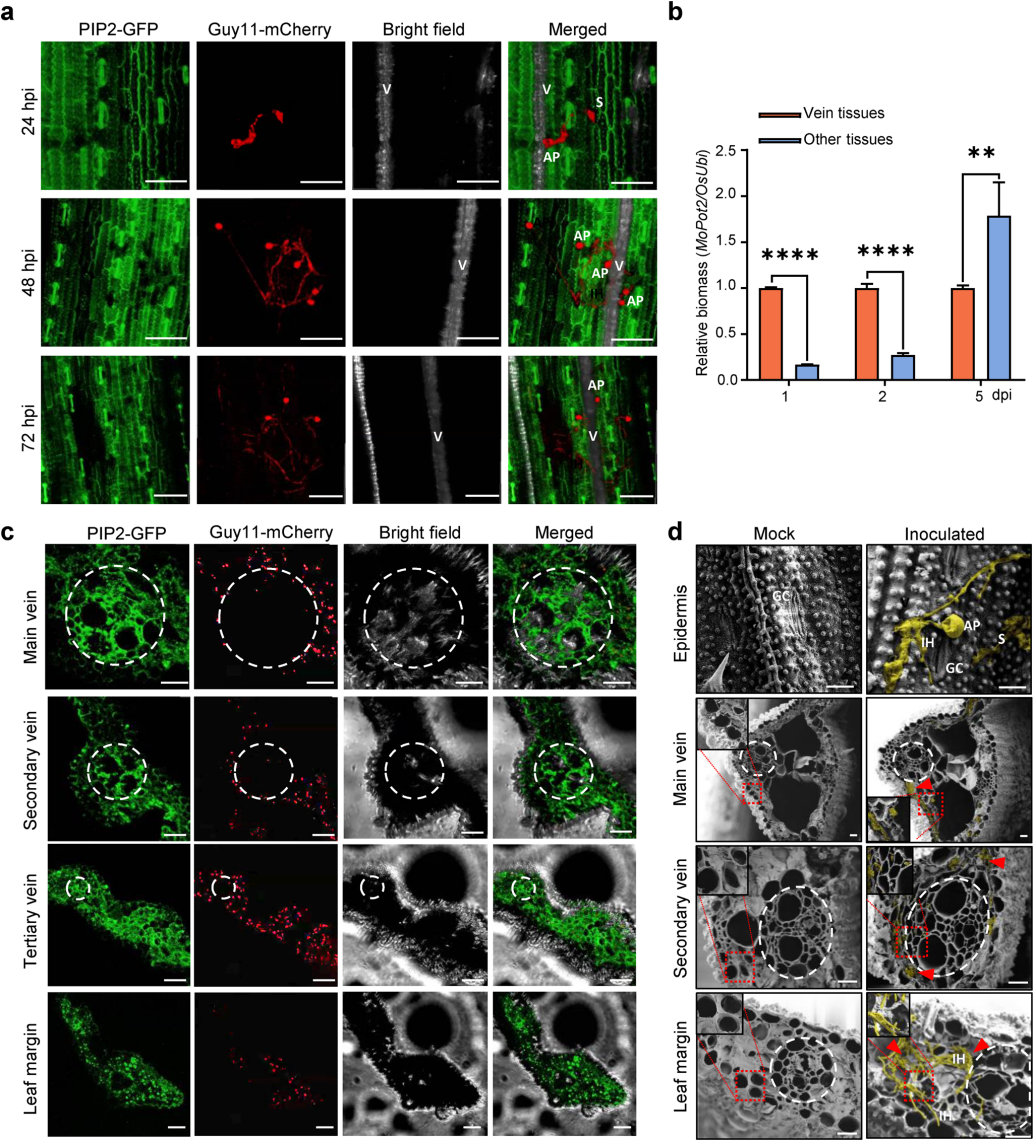
Rice vascular tissue exhibits a strong immune response to *M. oryzae* infection. a) Representative confocal micrographs show that *M. oryzae* targets rice leaf veins. The mCherry‐labeled *M. oryzae* strain Guy11 primarily targeted rice leaf veins. The rice cell plasma membrane was labeled with PIP2‐GFP to visualize cell profiles. The rice leaves were spray‐inoculated with *M. oryzae* spores at a concentration of 5 × 10^5^ spores mL^−1^. The infection process was observed with a confocal microscope at indicated time points. V, vein; S, spore; AP, appressorium; IH, invasive hyphae. Scale bars, 20 µm. b) Relative fungal biomass in vein and the rest of the tissues following the infection process. The fungal biomass was determined by qPCR of *MoPot2* gene against *OsUbi1* gene in *M. oryzae* and rice, respectively. The experiment was repeated three times with similar results. Each experiment used five infected rice leaves. The leaf tissues were separated as “vein tissue” and “other tissue”. ** represents the significance between the two samples by Student's *t*‐test at *p* < 0.01 and *p* < 0.0001(****), *n* = 5 leaves. c) Confocal microscopic observation of the distribution of *M. oryzae* hyphae in rice leaves. The infective hyphae were observed at lesion edge of rice leaf at 4 dpi. The mCherry‐labeled Guy11 spores were used to infect the rice leaves at a concentration of 5 × 10^5^ spores mL^−1^. The rice cell plasma membrane was labeled with PIP2‐GFP to visualize cell profiles. The circles indicate the vascular tissues. Scale bars, 20 µm. d) The infection process of *M. oryzae* hyphae in rice leaves. The infection process was observed with SEM at 48 hpi in different rice leaf tissues. The circles indicate the vascular tissues. The red square area was enlarged to view the fungal hyphae. Red arrowheads indicate the infected area with fungal hyphae. Yellow color indicates the invasion hyphae of *M. oryzae*. Red frame indicates magnified invaded areas. V, vein; S, spore; AP, appressorium; IH, invasive hypha; GC, Guard cell. Scale bars, 10 µm.

Given the indications suggesting vein tissues as the likely battleground during the interaction between *M. oryzae* and rice, our investigation delved into the spatial dynamics of infection. We employed a *35S:PIP2‐GFP* plant expressing GFP to label plasma membranes and the Guy‐11‐mCherry strain constitutively expressing mCherry in fungi for infection tracking. Initially, we focused on fungal invasion on the leaf surface at 24, 48, and 72 hpi. Surprisingly, we found that the vein or near vein regions were the main targets of *M. oryzae* (Figure **3**a; Video S1, Supporting Information). This observation was unexpected, considering veins consist of vascular tissues. To further verify this observation, we defined three possibilities of spores’ location that are in the middle of veins, near veins, and on veins (Figure S5a, Supporting Information). The confocal microscope images showed that the spores primarily targeted rice leaf veins for penetration although they were randomly distributed on the leaves (Figure S5b, Supporting Information). We counted the position of the appressorium, where 39.47% of the spores directly penetrated the veins (Figure S5c, Supporting Information).

Further, we assessed the relative fungal biomass in vein and non‐vein tissues at 1, 2, and 5 dpi. The results indicated that fungal biomass primarily accumulated in veins at 1 and 2 dpi, with no significant increase observed at 2 and 5 dpi, suggesting limited proliferation in vein tissues. In contrast, fungal biomass notably increased in non‐vein tissues at 5 dpi, supporting the notion that the vein tissues remained largely uninfected (Figure 3b). To substantiate this result, further investigation into the distribution of fungal hyphae within the skirt region of the infection site at 5 dpi revealed that while the fungi invaded most tissues, they could not penetrate the mestome sheath at the center of the midvein, including the main vein, secondary vein, and tertiary vein. Conversely, the leaf margin exhibited severe infection (Figure 3c), indicating that the mestome sheath effectively resisted *M. oryzae* invasion. Additionally, we employed SEM to validate our fluorescence observations. The SEM images revealed fungal spores mainly penetrating the epidermal region near the veins. Consistent with fluorescence observations, invasive hyphae were absent in the main and secondary veins but present in the leaf margin (Figure 3d). These findings collectively underscore the critical role of vascular tissues in resisting *M. oryzae* infection in plant leaves.

### 2.5 Phytohormone Signaling Pathways do not Show Cell‐Specific Responses

Phytohormones play a pivotal role in the defense against pathogen infections. Among them, salicylic acid (SA) and jasmonic acid (JA) are particularly involved in disease resistance. By analyzing the gene expression patterns related to these two hormones' signaling pathways, we found that they did not exhibit cell‐type‐specific responses, showing similar expression patterns across all cell types. Notably, the activation of hormone signaling pathways was dramatically triggered at 24 and 48 hpi, but not at 12 hpi, suggesting that hormone signaling did not promptly respond to the *M. oryzae* infection at the early infection stage (Figure S6, Supporting Information). In particular, the activation of SA signaling is required for blast resistance in rice.^[^ ^30^ ^]^ However, the expression of SA‐responsive genes *OsPR1b*, *OsPR1*, *OsPR10a*, *OsPBZ1*, and *OsWRKY45* showed no significant induction at 12 hpi (Figure S6a and Table S4a, Supporting Information). In contrast, *OsJAZ1* was slightly down‐regulated at 12 hpi (Figure S6b and Table S4b, Supporting Information). Only three JA biosynthesis genes (*OsAOS*, *OsLOX9*, and *OsLOX8*) were differentially regulated in most cell types at 24 and 48 hpi, indicating that JA synthetic pathway does not exhibit cell‐specific expression either (Figure S6b and Table S4b, Supporting Information). It is known that JAZ proteins act as repressors of JA signaling. The down‐regulation of *OsJAZ1* would theoretically lead to JA signaling activation. Indeed, it has been observed that JA signaling antagonizes SA signaling at the early infection stage in the *M. oryzae* and rice interaction.^[^ ^30^ ^]^ Following the infection, SA signaling was initiated, but JA signaling was inhibited at 24 hpi, and the inhibition was subsequently released at 48 hpi. Considering that JA signaling activation is required for resisting necrotrophic pathogen infections, the transcriptomic switch observed in plant cells from 24 to 48 hpi may be attributed to the developmental switch of *M. oryzae* from biotroph to necrotrophy.

### 2.6 Rice Cells Exhibit Active PTI Responses During Infection

**Figure 4:**
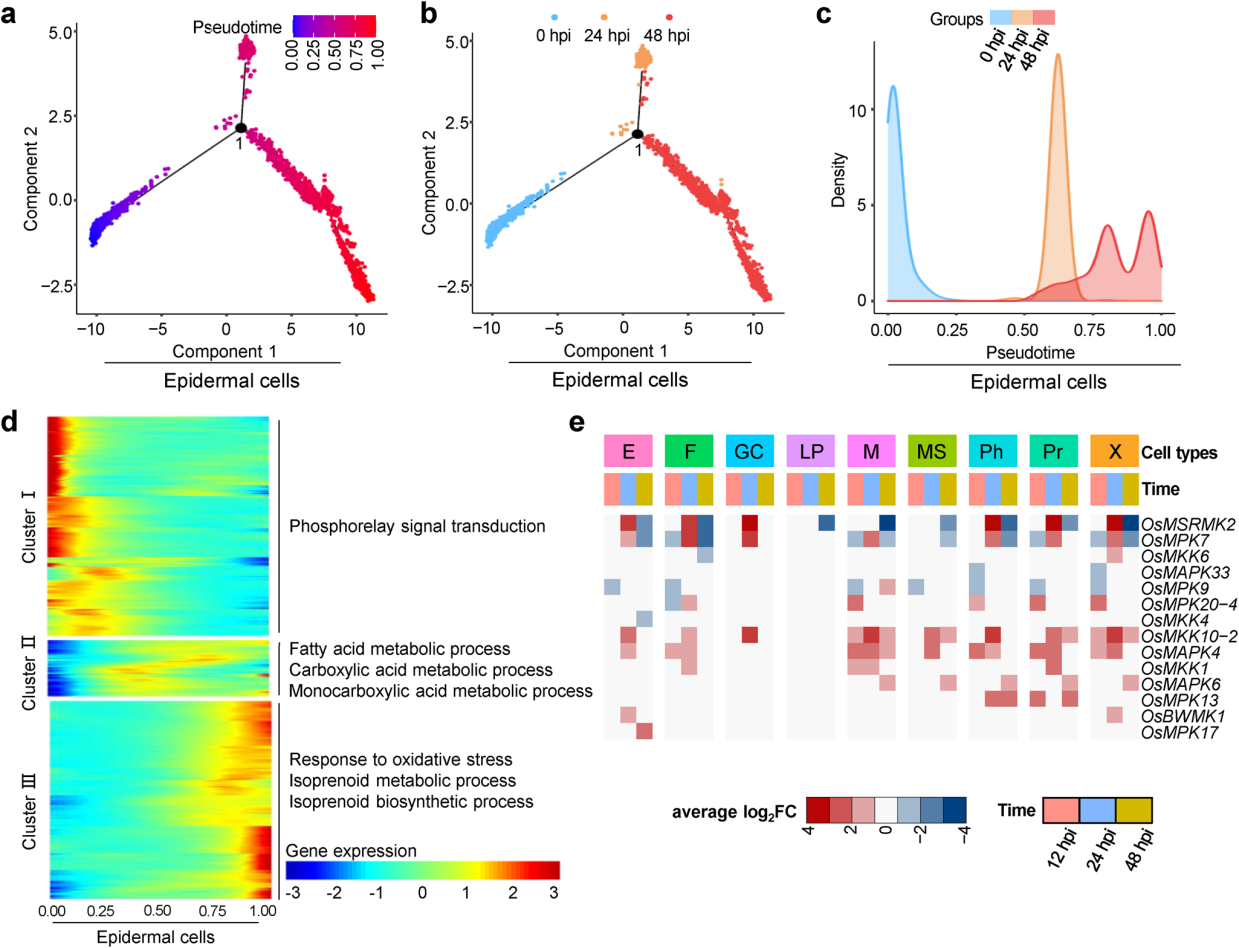
Trajectory curves of rice epidermal cells in response to blast infection. a) The trajectory curve of epidermal cells from the infected rice leaves. 1714 genes (*q* value < 0.01) from infected samples were used to construct the trajectory curve. Each dot represents a single epidermal cell. The color of a dot indicates its pseudotime value. Number “1” indicates the branching points. b) Trajectory curves of epidermal cells from mock, 24, and 48 hpi samples. Epidermal cells at different time points were placed in chronological order along the trajectory curve. Each dot represents a single cell. The color indicates the infected cells at 0, 24, and 48 hpi. c) The curve uniformity between pseudotime and infection time during *M. oryzae* infection. The shift of pseudotime was set from “0” to “1” in the epidermal cells. The color indicates the infected samples at 0, 24, and 48 hpi. d) The cellular biological processes enriched in epidermal cells at different pseudotime values. The heatmap shows the expression levels of pseudotime‐dependent genes in the trajectory of epidermal cells. The genes were grouped into three populations based on hierarchical clustering. Representative gene ontology (GO) terms enriched in each population are listed. e) Cell‐type‐specific expression of MAPK signal pathway‐related genes during *M. oryzae* infection. Heatmaps show expression of MAPK signal pathway‐related genes from rice in each annotated cell type. The DEGs expression levels are indicated by colored average log_2_FC. *p* adj < 0.01, pct.1>0.1 for up‐regulated genes, and pct.2 > 0.1 for down‐regulated genes, |average log_2_FC|>1.0. M, Mesophyll; X, Xylem; F, Fiber; Pr, Procambium; MS, Mestome sheath; E, Epidermis; GC, Guard cell; Ph, Phloem; LP, Large parenchyma.

The trajectory curve assay is a valuable method for analyzing the heterogeneous cellular responses to a given stimulus.^[^ ^7^, ^31^ ^]^ Initially, epidermal cells engage pathogens directly. To examine the immune processes during *M. oryzae* infection, we focused on epidermal cells. Using the Monocle2 analysis method,^[^ ^32^ ^]^ we constructed a trajectory curve for 1714 genes in epidermal cells (Table S5a,b, Supporting Information). Concurrently, we assessed the immune response continuity of epidermal cells in mock and *M. oryzae*‐treated samples by constructing trajectory curves. The results indicated that *M. oryzae*‐treated cells followed a trajectory curve along pseudotime (from “0” to “1”), which correlated with the curve constructed for all epidermal cells to help understand the infection process (Figure **4**a,b). In contrast, mock samples did not align with the pseudotime‐dependent trajectory curve (Figure S7a,b, Supporting Information). Furthermore, unlike mock samples, epidermal cell populations could be accurately correlated with the infection time along pseudotime (Figure 4c; Figure S7c, Supporting Information). These findings demonstrate that the trajectory curve based on epidermal cells delineates fungal infection in a spatially continuous manner.

Subsequently, we conducted GO term enrichment analysis along pseudotime in epidermal cells following *M. oryzae* treatment. The top 2000 DEGs were identified and subjected to GO analysis. Cellular processes were primarily enriched at three pseudotime values (Figure 4d; Table S5c, Supporting Information). At the outset of pseudotime, phosphorelay signal transduction was significantly enriched, likely reflecting active phosphorylation reactions during PTI. In the middle of pseudotime, fatty acid metabolic process and carboxylic acid metabolism pathways were enriched, possibly contributing to the enhanced antimicrobial activity.^[^ ^33^ ^]^ Toward the end of pseudotime, response to oxidative stress and isoprenoid biosynthetic process were enriched, which may be attributable to ROS production and active metabolism processes.^[^ ^34^ ^]^ In addition, PTI signal pathway‐related genes were commonly induced in all cell types except large parenchyma (Figure S8 and Table S6, Supporting Information).

Given the potential activation of PTI in epidermal cells (Figure 4d), we investigated the cell‐specific response to the mitogen‐activated protein kinase (MAPK/MPK) cascade, a crucial intercellular signaling pathway downstream of PTI.^[^ ^35^ ^]^ We analyzed the DEGs of MAPK in all cell types. The results revealed that MAPKs were scarcely induced at 12 hpi but differentially induced at 24 hpi. Among these MAPKs, OsMSRMK2 and OsMPK7 serve as negative regulators of rice blast disease, whereas OsMKK1, OsMAPK4, and OsMAPK6 positively regulate rice disease resistance.^[^ ^36^, ^37^, ^38^ ^]^ Notably, MAPK expression was not induced in larger parenchyma cells and slightly induced in epidermal cells at early infection stage (Figure 4e; Tables S5d and S6, Supporting Information). Further KEGG analysis demonstrated that the MAPK pathway was specifically enriched and up‐regulated at 24 hpi but down‐regulated at 48 hpi in epidermal cells (Figure S9a,b and Table S5e,f, Supporting Information). Guard cells, as a specialized epidermal cell type, serve as the entry point for many pathogens. We also analyzed MAPKs in guard cells, revealing enrichment of the MAPK pathway at 24 hpi, although it was undetectable at 48 hpi (Figure S9c,d and Table S5g,h, Supporting Information). These results indicated that following hyphal development in leaf tissues, rice cells undergo active PTI responses, with epidermal cells exhibiting activity only during the early infection stage at 24 hpi, while guard cells exhibiting activity at 24 hpi, but not 48 hpi, suggesting that following the infection of fungal hyphae, different cell types activated their PTI responses.

### 2.7 Rice Leaves Exhibit a Longitudinal Polarity in Immune Response

**Figure 5:**
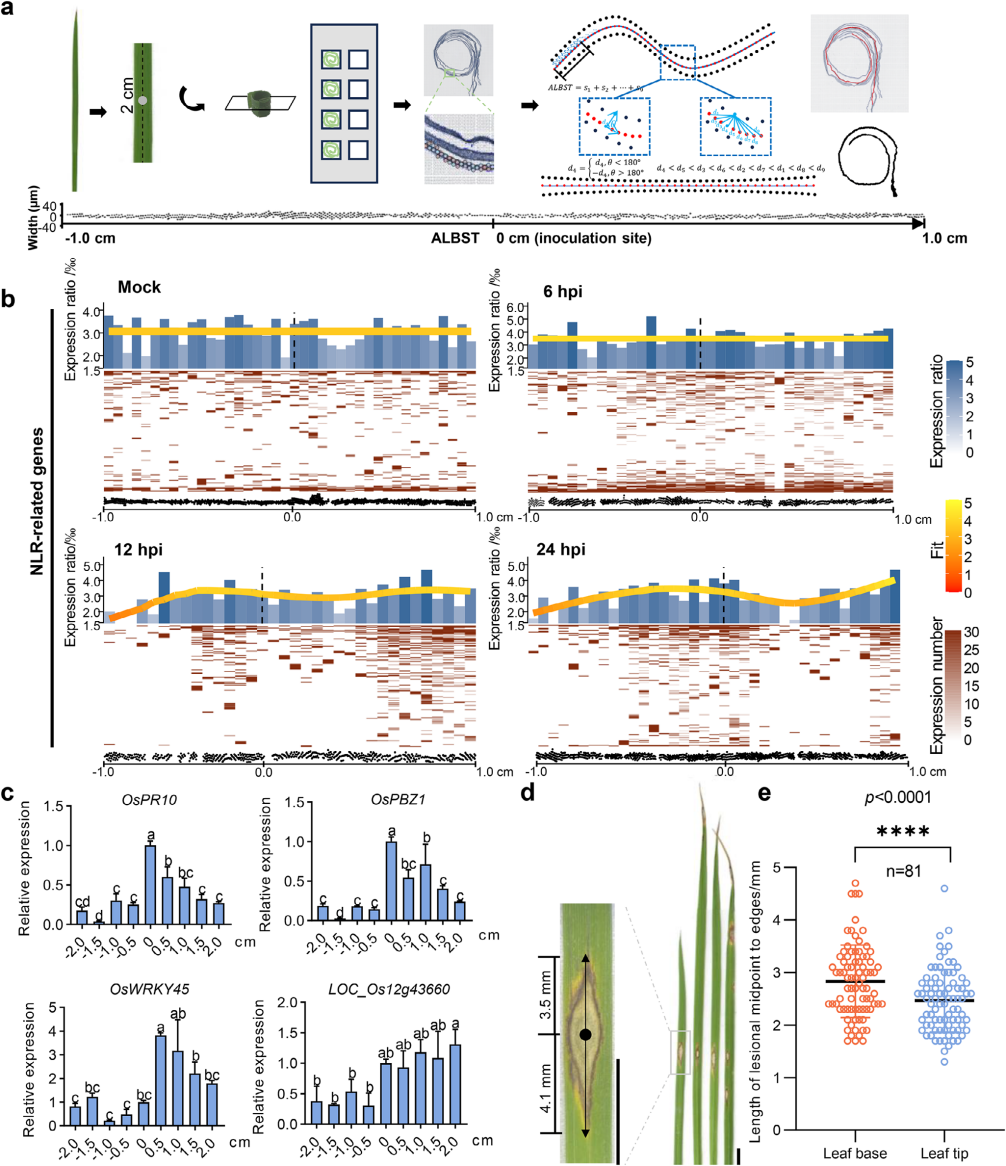
Spatial transcriptome revealed dynamic expression of immune receptor genes. a) Schematic diagram of spatial transcriptomic RNA sequencing (stRNA‐seq) for rice leaves. Rice leaf fragments (2 cm) were rolled up and sliced longitudinally to fit in the sequencing chip (0.68 cm × 0.68 cm) for stRNA‐seq. The UMI counts in the arrays were matched to the physical position of leaf tissues. To visualize the gene expression linearly, the “rolled” spots assigned to the leaf fragments were reshaped to a horizontal curve by the program LOONG v1.0 (described in Material and Methods). The position of “0 cm” is the inoculation site. Each assay has two biological replicates. b) Heatmap and barplot assays were used to characterize the numbers of detected NLR‐related immune receptor genes. The leaf fragment (2 cm) was equally divided into 40 plots. Barplot shows the expression ratio of all detected genes based on heatmaps (lower panels) at different plots. The fit lines represent a fitted expression ratio value calculated by R function “mgcv::gam”. The ratio of detected gene numbers was curve fitted for the leaf fragments at 0, 6, 12, and 24 hpi. “0 cm” is the infection site. Expression ratio indicates the number of detectable NLR genes versus the number of total detectable genes in each leaf fragments. “Dotted line” indicates the infection site. c) The relative expression levels of defense responsive genes *OsPR10*, *OsPBZ1, OsWRKY45*, and PRR gene *LOC_Os12g436e60* in rice leaf fragments. The leaves of two‐week‐old plants were inoculated with *M. oryzae* spores. Four centimeters of leaf fragments centering the inoculation site were sampled for RT‐qPCR assay at 24 hpi. *OsActin1* was used as the internal reference gene. Letters indicate the significant difference between the expression levels at different sites by one‐way ANOVA at *p* < 0.05 (*n* = 3 biological repeats). d) Disease symptoms of rice leaves infected by *M. oryzae*. Disease symptoms were observed after spray‐inoculated with *M. oryzae* spores at a concentration of 5 × 10^5^ spores mL^−1^. The photos were taken at 5 dpi. Scale bars, 4 mm. e) Statistical analysis of lesion length from the lesion midpoint toward two different directions (base and tip). The leaves were sampled from protected paddy fields. “****” represents the significance between the two samples by Student's *t*‐test at *p* < 0.0001.

Although single‐nucleus RNA sequencing (snRNA‐seq) holds great potential in identifying gene expression across various cell types, achieving a gene expression profile at a spatial level remains challenging. Recently, spatial transcriptome RNA sequencing (stRNA‐seq) techniques have emerged and been utilized in numerous studies.^[^ ^39^, ^40^ ^]^ In this study, we employed stRNA‐seq to investigate spatial transcriptomics in rice leaves treated with *M. oryzae*, aiming to further dissect the infection progression. Due to space constraints on the chips (0.68 cm × 0.68 cm), we rolled up leaf fragments to fit them for subsequent stRNA‐seq assays (Figure **5**a). To linearize the gene expression profile from the rolled leaves, we developed the LOONG program (v1.0) (https://github.com/ltpr31/LOONG/) to sort and rearrange selected spots (gene arrays) along a unidirectional spatial trajectory. Leaf segments covering spots were chosen from the spatial transcriptome data using BSTViewer. Subsequently, the images and coordinates of the spots were loaded into LOONG, allowing us to virtually straighten the curly leaf segment‐associated spots onto auxiliary lines (red) (Figure 5a). We successfully obtained an average of 942 detected genes per spot (L6). Additionally, an average of 1564 unique molecular identifiers (UMIs) were captured for each spot (L6) (Figure S10, Supporting Information).

To visualize gene expression, we divided the 2.0 cm‐long leaf fragment into 40 plots. Barplot and heatmap assays were utilized to demonstrate the expression ratio of NLR‐encoding genes (Figure 5b). Notably, the expression ratio did not increase or even slightly decreased toward the leaf base ≈0.5 cm distant from the infection site labeled as “0 cm” at 12 hpi. Surprisingly, at 24 hpi, NLRs expression ratio slightly decreased toward the leaf base ≈1.0 cm distant and slightly increased toward the leaf tip near 1.0 cm from the infection site labeled as “0 cm”. (Figure 5b; Table S7, Supporting Information). These data indicate that immune signals exhibit polarity along the longitudinal axis, with younger tissue (toward the leaf tip) displaying stronger immune responses. Additionally, the expression ratios of PRRs were slightly repressed in leaves infected by *M. oryzae* (Figure S10b and Table S8, Supporting Information).

To validate this observation, we employed RT‐qPCR to examine the expression levels of key defense marker genes at different distances from the inoculation site. Consistent with our spatiotemporal results, *OsPR10*, *OsPBZ1*, *OsWRKY45*, and the PRR gene *LOC_Os12g43660* were significantly induced toward the leaf tip but not the leaf base (Figure 5c). Moreover, we also measured the length of disease lesions from midpoint to two directions (Figure 5d,e). The result indicated that the lesions toward the leaf tip were shorter than that of the leaf base. It also suggested that PRRs and NLRs contributed to the longitudinal resistance.

### 2.8 Potassium Transport and Isoprenoid Biosynthesis were Active in Procambium Cells

**Figure 6:**
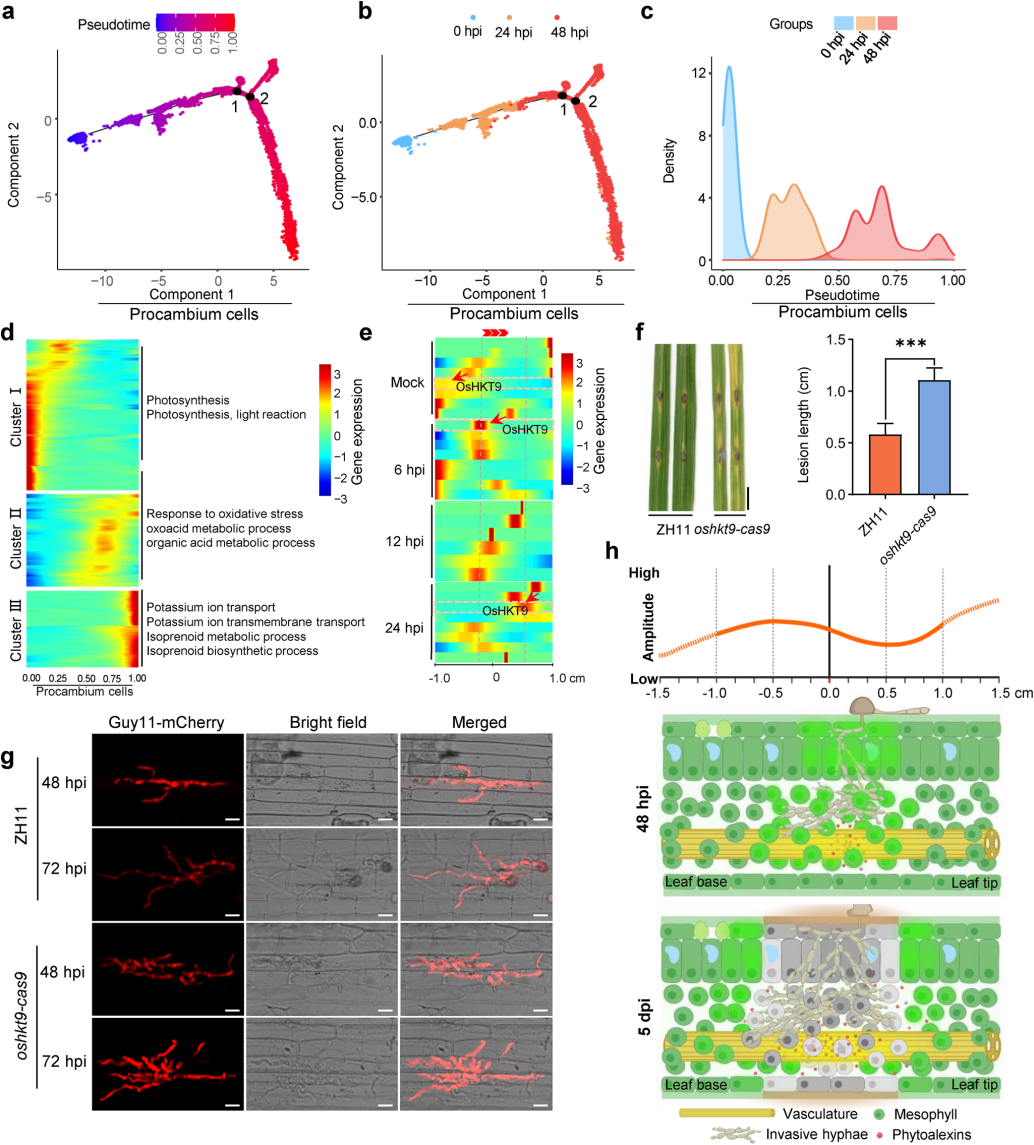
*OsHKT9* is required for blast disease resistance in rice. a) The trajectory curve of procambium cells from the infected rice leaves. 1764 genes (*q* value <0.01) from infected samples were used to construct the trajectory curve. The color of a dot indicates its pseudotime value. Each dot represents a single epidermal cell. b) Trajectory curves of procambium cells from mock, 24, and 48 hpi samples. Each dot represents a single procambium cell. The color indicates the infection time. c) The curve uniformity between pseudotime and infection time of *M. oryzae*. A shift of pseudotime was set from “0” to “1” in the procambium cells. The color indicates the infected samples at 0, 24, and 48 hpi. d) Cellular processes were enriched in procambium cells that have different pseudotime values. The heatmap shows relative expression levels of pseudotime‐dependent genes in the trajectory of procambium cells. The processes were grouped into three populations based on hierarchical clustering. Representative gene ontology (GO) terms enriched in each population are highlighted. e) Trajectory heatmap of the spatial expression of *OsHKT9*. *OsHKT9* expression was examined by stRNA‐seq with dynamically spatial value in infected rice leaf fragments. The red arrowheads point to the dynamic expression of *OsHKT9* at different leaf positions during infection. f) *OsHKT9* mutant plants exhibited reduced disease resistance to *M. oryzae*. Left panel, disease symptoms by punch inoculation with *M. oryzae* spores; Right panel, measurement of the lesion length. “***” represents *p* < 0.001 (Student's *t*‐test, *n* = 4 biological replicates). g) Micrographs of rice sheath inoculated with *M. oryzae*. *M. oryzae* strain Guy11‐mCherry was used to observe the infection. Images were taken at 48 and 72 hpi. Scale bars, 10 µm. h) The model of spatiotemporal landscape of the interaction between rice and *M. oryzae*. The vascular tissues mount defenses by producing phytoalexin and expressing immune proteins at early infection stages (prior to 48 hpi) and consequently prevent the pathogen from colonizing vascular tissues. In the longitudinal direction, the neighboring cells surrounding the inoculation site exhibited strong immune responses by activating potassium ion transport in procambium cells, which restricts further pathogen infection of the tissues. The immune strength is stronger toward the leaf tip than that of the leaf base. The curve indicates the immune output at the longitudinal axis. Solid line represents the detected spatial defense gene expression. Gray cells showing the lesion. Greener cells show the local acquired resistance (LAR).

Given that the procambium cells likely play crucial role in constraining fungal spreading (Figure 2g; Figure S4, Supporting Information), our study aimed to investigate gene expression patterns along the longitudinal axis of leaves. We constructed trajectory curves of procambium cells for both mock and *M. oryzae*‐treated samples (comprising 1764 genes). Notably, unlike mock samples, cells treated with *M. oryzae* exhibited a chronological order along the trajectory curve at various time points (**Figure** **6**a–c; Figure S7a–c and Table S9a,b, Supporting Information), indicating a continuous response in procambium cells. To further elucidate the dynamics of procambium cell responses during infection progression, we conducted the GO enrichment analysis. Among the functional annotation enrichment analysis, 42 bioprocesses were notably enriched at the initial phase of the curve (close to a pseudotime value of 0) (Table S9c, Supporting Information). These genes primarily clustered in processes such as photosynthesis and light reaction (Figure 6d). Conversely, processes such as “response to oxidative stress,” “oxoacid metabolic process,” and “organic acid metabolic process” were predominant in the middle phase of the curve, reflecting active immune responses to pathogen infection in the vicinity of procambium cells. Remarkably, potassium transport and isoprenoid biosynthesis emerged as prominent biological processes, with upregulated isoprenoid biosynthesis potentially facilitating the activation of isoprenoid‐type phytoalexin biosynthesis, including momilactone A (Figure 6d; Table S9c, Supporting Information).

In line with spatial expression patterns observed for *PRRs* and *NLRs*, genes associated with ion transport were spatially induced by *M. oryzae* (Figure S11 and Table S10, Supporting Information). Given the significant activation of potassium transport pathways in procambium cells at a pseudotime of “1.0,” and the recognized role of potassium in blast resistance in rice,^[^ ^41^ ^]^ we focused our investigation on potassium transport‐related genes. Notably, further analysis of stRNA‐seq data revealed dynamic induction of potassium transport genes during the infection process (Figure 6e). For instance, the gene *OsHKT9* (*LOC_Os06g48800*) was induced at the inoculation site at 6 hpi. However, at 24 hpi, it exhibited high expression levels at 0.5 cm toward the leaf tip, while expression was undetected at the opposite site to the leaf base and the inoculation site (Figure 6e). Expression was also not detected at the inoculation site at 12 hpi (Figure 6e). These findings suggest that following successful penetration of the epidermis by the fungus, *OsHKT9* or similar genes were suppressed by the pathogen, facilitating proliferation within leaf tissue. However, expression in distal tissues toward the leaf tip was either normal or slightly induced, thus constraining further pathogen proliferation.

To investigate the role of OsHKT9 in rice blast resistance, we inoculated *os* *hkt9* knockout plants with *M. oryzae* spores. Compared to ZH11 (wild‐type, WT), *os* *hkt9‐cas9* mutants showed longer lesions in leaves (Figure 6f). Subsequent sheath‐inoculation experiments with *M. oryzae* strain Guy11 expressing mCherry in both ZH11 and *oshkt9* mutant revealed that fungal hyphae developed more extensively in the first or second infected cell in *oshkt9* mutant than in WT plants (Figure 6g). These results indicate that *OsHKT9* may restrict pathogen spreading, ultimately leading to shorter lesions toward leaf tip (Figure 5d).

## 3 Discussion

Single‐cell RNA sequencing (scRNA‐seq) has been extensively utilized to investigate gene expression profiles at single‐cell resolution in plants.^[^ ^42^, ^43^, ^44^ ^]^ This technique has also recently been applied to study plant immune responses,^[^ ^7^, ^31^, ^45^ ^]^ providing a comprehensive view of transcriptomes and dynamic gene expression following pathogen infection in plants. Using snRNA‐seq, we identified nine distinct cell populations from transcripts obtained from 236 708 cell nuclei derived from the rice leaves, encompassing all major cell types (Figure S2b,c, Supporting Information). To investigate gene expression in a spatial landscape, we also employed a recently developed technique, spatial RNA sequencing (stRNA‐seq), to analyze plant cell transcriptomes under *M. oryzae* infection. A similar technique has recently been employed to study host‐bacteria‐fungi interactomes in *Arabidopsis* leaves and plant development.^[^ ^40^ ^]^ With this spatial‐temporal transcriptome atlas, we were able to stereoscopically dissect the fungal infection process.

Pathogens colonize various plant tissues, with a notable preference for vascular tissue. *Xanthomonas campestris* pv. *campestris*, for instance, exhibits a preference for vascular invasion, colonizing the interior of epidermal cells, the apoplast, and the xylem vessels within the vasculature.^[^ ^46^ ^]^ The destructive cotton wilt pathogen *Verticillium dahliae* infects and proliferates within the xylem of cotton plants,^[^ ^47^ ^]^ thereby disrupting water and nutrient transport through the vasculature. During *M. oryzae* infection, our observations indicate that fungal germ tubes specifically target rice leaf veins for infiltration (Figure 3). The vascular system serves as a bidirectional transport corridor, facilitating the conveyance of organic and inorganic ions as well as water to all cells. The nutrient‐rich nature of the vascular system likely renders it a primary target for pathogen exploitation. Consequently, the vascular region emerges as a pivotal battleground in the interaction between plants and pathogens.

Indeed, Wang et al. identified an ER‐localized calcium‐permeable channel, WeiTsing, which facilitates the pericycle's ability to hinder *Plasmodiophora brassicae* colonization in the stele.^[^ ^48^ ^]^ Similarly, Tang et al. discovered that the vascular‐enriched mean expression of NLRs, especially the TNLs and RNLs, is heightened during fungal pathogen *Colletotrichum higginsianum* infection in *Arabidopsis*, indicating that vascular immunity plays an essential role in disease resistance.^[^ ^31^ ^]^ Upon *M. oryzae* infection, there was a slight induction of PRRs and no significant induction of NLRs in epidermal cells. Expression of some PRRs and NLRs decreased in mesophyll cells. Many other cells, especially the mestome sheath cells, expressed a considerable number of PRRs and NLRs (Figure 2b), suggesting their active involvement in immunity during infection. Given that PRRs‐mediated immune response is pivotal in thwarting pathogen invasion at the initial stage, the subdued responses in epidermal and mesophyll cells may account for why *M. oryzae* could successfully invade the epidermis and establish infection.

Surprisingly, we found that procambium cells significantly enriched key phytoalexin biosynthesis gene transcripts, especially at 48 hpi. Momilactone A serves as the major phytoalexin in rice. Measurement of momilactone A levels in vascular and non‐vascular tissues post‐pathogen infection revealed primary accumulation in vascular tissues, corroborating the snRNA‐seq data. Thus, similar to *Arabidopsis* challenged with *C. higginsianum*,^[^ ^31^ ^]^ vascular‐related tissues serve as the key battleground in rice. However, unlike the enhanced expression of TNLs in *Arabidopsis* upon *C. higginsianum* infection, rice employs a strategy of accumulating phytoalexins and PRRs and NLRs in procambium cells against *M. oryzae* infection. Consequently, *M. oryzae* fails to colonize vascular tissues, although it is preferring to infect veins (Figure 6h). Notably, why phytoalexin biosynthesis genes were exclusively induced in procambia cells and whether the phenomenon is specific to monocot plants are worth of further investigation.

Nevertheless, the progression of epidermal cell responses along the pseudotime indicates activation of immune signaling at the infection site (Figure 4d). As pseudotime approaches values of 0 and 0.5, there is an enrichment of phosphorelay and oxidative stress signals, suggesting early‐stage defense activation. Indeed, the PRRs‐mediated immune response is observed across all cell types except for large parenchyma cells (Figure S8, Supporting Information), evidenced by the induction of MAPK cascade genes; however, they exhibit reduced responsiveness at 12 hpi, particularly in epidermal cells (Figure 4e). Guard cells within the epidermis actively close to prevent pathogen entry.^[^ ^49^, ^50^ ^]^ For example, a transcriptome shift occurs in *Arabidopsis* guard cells during *C. higginsianum* infection, with multiple pathways contributing to stomatal closure.^[^ ^31^ ^]^ Induction of MAPK‐cascade genes is also observed in guard cells at 24 hpi during *M. oryzae* infection (Figure 4e; Figure S9c, Supporting Information). However, stomatal immunity may not be crucial for rice plants to defend against *M. oryzae* infection, as the pathogen penetrates leaf interiors via appressorium penetration through the epidermis. Thus, MAPK activation in rice guard cells at the early stage may not aid in resisting *M. oryzae* invasion. Notably, the widespread expression of MAPKs in most cell types at 24 and 48 hpi suggests a comprehensive PTI response in leaf tissues, likely due to direct contact with fungal hyphae.

It is also worth mentioning that the dynamic changes in jasmonic acid (JA)/salicylic acid (SA) signaling occur during infection. We previously observed that JA signaling was activated and SA signaling was suppressed at the early infection stage, but both pathways were upregulated at later infection stages.^[^ ^30^ ^]^ We proposed that the suppression of SA signaling at the early stage led to the successful infection of *M. oryzae*. Here, the snRNA‐seq data for JA and SA expression patterns supports our previous observation. Interestingly, they exhibited uniform expression in all cell types, indicating that SA/JA signaling is not cell‐type specific.

Recent advances in stRNA‐seq have enabled the investigation of cell‐specific developmental processes in both animal and plant sciences.^[^ ^12^, ^51^ ^]^ This technique has proven advantageous in addressing issues of spatial heterogeneity. For instance, Xia et al.^[^ ^39^ ^]^ demonstrated subtle yet significant transcriptomic variances between upper and lower epidermal cells of *Arabidopsis* leaves, as well as cell‐type‐specific gene expression gradients from the main vein to the leaf edge. Similarly, developmental stages of rice leaves exhibit gradient expression patterns along the longitudinal axis. Li et al.^[^ ^10^ ^]^ observed a gradual transition in transcriptomes from the leaf base to the tip. We hypothesize that identical cell populations within leaves exhibit varying responses along the longitudinal axis upon pathogen infection. As anticipated, our findings reveal a polarized expression pattern of immune genes during blast infection. Proximal to the infection site, immune genes are highly induced, whereas expression diminishes in the immediate vicinity of the infection. Surprisingly, immune gene expression increases in cells further from the infection site, particularly toward the leaf tip rather than the base. These results suggest that younger cells exhibit a stronger immune response along the longitudinal axis to pathogen infection.

The trajectory curve of procambium cell responses indicated that the cells exhibit spatial expression differences (Figure 6b). Notably, at the end of the pseudotime (assigned for infection process from beginning 0.0 to the end of infection 1.0) in trajectory curve, in addition to the active biosynthesis of isoprenoid phytoalexins, there was enriched gene expression of potassium ion transport (Figure 6d). It is known that a high potassium status in plants could decrease the incidence of many diseases or pests.^[^ ^52^ ^]^ Similarly, high potassium levels enhance disease resistance against *M. oryzae* in rice.^[^ ^41^ ^]^ Among the potassium ion transport‐related genes, *OsHKT9* exhibited spatially dynamic expression, being expressed at the infection site at 6 and 24 hpi, but by 12 hpi, it could not be detected there. Instead, the expression was induced in cells in close proximity to the ultimate lesion edge, about 0.5 cm away from the inoculation site. Since OsHKT9 is required for disease resistance (Figure 6f), the expression of the potassium transport‐encoding gene may restrict the further spread of the pathogen. Pathogen infection can lead to specific lesions, which often serve as signs of disease symptoms. *M. oryzae* infection typically forms diamond‐shaped lesions on rice leaves. The differential immune responses along the longitudinal axis may be associated to the developmental gradients in the leaf and the vascular immunity across the cross‐section are likely reasons for explaining this specific lesion shape (Figure 6g).

Although the development of *M. oryzae* hyphae in host tissue may undergo a progressive biotrophic/necrotrophic stage, further infection along the longitudinal axis was observed to be attenuated between 5 to 7 days. Our data suggest that cell‐specific immunity along the longitudinal axis prevents continuous infection, which may subsequently compel the hyphae to transition into the necrotrophic stage. Jacob et al. proposed that local acquired resistance (LAR) might contribute to halting the pathogen at the infection site.^[^ ^53^ ^]^ According to their hypothesis, LAR is activated by locally infected cells programmed for death due to a hypersensitive response. Our results reveal that PRRs, NLRs, and many other defense‐related genes were indeed induced in many other cells surrounding the infection site during the early stages of infection (12 and 24 dpi). Although they hinder pathogen further infection, the mechanisms underlying their induction require further investigation.

In summary, we utilized snRNA‐seq and stRNA‐seq techniques to dissect the process of *M. oryzae* infection in rice leaf tissue, providing comprehensive insights into the cellular composition of rice leaves and spatial variations in cell responses during blast infection. The temporal and spatial transcriptome in the specific cell types of rice leaves enables us to precisely pinpoint the key immune cells and the genes (Figure 6h). Overall, plant vascular tissue appears to play a pivotal role in impeding pathogen infection through heightened immunity compared to other cells, rendering it a promising target for enhancing disease resistance in crops. Furthermore, LAR may be indispensable in curbing pathogen spread longitudinally, bolstering the notion of LAR's presence in non‐infected cells. Our study thus offers a dynamic and multidimensional understanding of fungal infection in plant leaves.

## 4 Experimental Section

### Plant Growth and Pathogen Inoculation

Rice (*Oryza sativa* subsp. *Japonica* cv. Nipponbare) seedlings were grown in a light incubator under 14/10‐h light/dark conditions at 26–28 °C. The relative humidity is 80% and light intensity is 12 000 Lux. The *aslrk*‐KO lines were obtained from Du et al.^[^ ^24^ ^]^ and the *os* *hkt9*‐c*as9*, *LOC_Os10g22510‐cas9*, and *LOC_Os08g29809‐cas9* lines were obtained from BIOGLE GeneTech (China). *M. oryzae* strain Guy11 was cultured on oatmeal agar medium (oatmeal 40 g L^−1^, calcium carbonate 0.6 g L^−1^, vegetable juice 100 mL^−1^, agar 20 g L^−1^) under light at 28 °C. To prepare conidia, the mycelia were scraped and grown under light for two days. The conidia were harvested and re‐suspended in water. The conidial suspension was prepared with 0.02% Tween‐20 to a final concentration of 5 × 10^5^ conidia per mL for inoculation on rice leaves.

For *M. oryzae* inoculation, the leaves of rice seeding were inoculated with conidia following the methods described previously.^[^ ^54^ ^]^ Briefly, twenty leaves of two‐week‐old rice seedlings were spray‐ or punch‐inoculated with *M. oryzae* conidial suspensions at a concentration of 5 × 10^5^ conidia per mL in 0.02% Tween‐20. The inoculated rice seedlings were kept in the incubator under dark at 26 °C for 20 h. Then, the plants were grown under 14/10‐hour light/dark conditions at 26–28 °C. Finally, the leaves were collected for snRNA‐seq or statistical analysis for disease symptoms.

### Single‐Nucleus RNA Library Construction and Sequencing

The nuclei isolation procedure was modified from the manual of the CelLyticTM PN Isolation/Extraction Kit (Sigma). The inoculated rice leaves were ground in 1×NIBTA buffer (1×NIB, 1 mM dithiothreitol, 1×ProtectRNA RNase inhibitor, 1×cOmplete, ethylene diamine tetraacetic acid (EDTA)‐free Protease Inhibitor Cocktail, and 0.3% Triton X‐100) on prechilled plates, and the homogenates were transferred to 15‐mL tubes. The tubes were then shaken for 5 min at 4 °C and the lysates were filtered through 40‐µm strainers. The flow‐through was collected. The samples were centrifuged at 1260×*g* for 10 min. The pellets were re‐suspended in 4 mL of 1×NIBTA buffer. Lysates were carefully overlaid onto Percoll layers prepared by 80% Percoll solution (4 mL Percoll plus 1 mL NIBTA buffer) and were subjected to centrifugation at 650×*g* for 30 min. The nuclei band at the 1×NIBTA buffer and Percoll interface were gently collected in 10 mL of 1×NIBTA buffer. Then, the nuclei were centrifuged at 1260×*g* for additional 5 min. Nuclei pellets were collected and washed twice in 1×NIBTA buffer. The nuclei were further re‐suspended in PBS buffer containing 0.04% BSA to a final concentration of 2000 nuclei µL^−1^ for snRNA‐Seq.

The snRNA‐seq libraries were prepared as previously described^[^ ^55^ ^]^ with DNBelab C Series High‐throughput Single‐Cell RNA Library Preparation Kit (MGI, 940‐000047‐00). Sequencing was performed using MGI 2000, DNBSEQ T7, and DIPSEQ T1.

### Analysis of snRNA‐seq Data

To align the raw snRNA‐seq data, parseFqDev was used to filter raw sequencing reads from DIPSEQ‐T1. STAR (v.2.7.1a) was used to align filtered reads to *O. sativa* genome (release 7 of the MSU), which was downloaded from the Rice Genome Annotation Project database.^[^ ^56^ ^]^ PISA (v.0.12b) (https://github.com/shiquan/PISA) was then used to generate cell versus gene unique molecule identifier (UMI) count matrices. The percentage of aligned reads ranged from 90.74% to 94.75% across the samples.

The downstream analysis was conducted by deploying scripts modified from Seurat (v4.0).^[^ ^14^ ^]^ Read10x was used to load raw matrix files, and the function “CreateSeuratObject” was used to build individual Seurat datasets. To filter out low‐quality data, cells with less than 250 rice genes were discarded, and the genes present in less than three cells were not considered. The dataset was then normalized and clustered by “SCTransform” and “FindClusters”. Douletfinder workflow was employed to identify doublets during the single‐nucleus partition experiment.^[^ ^57^ ^]^ Function “DoubletFinder_v3” was used to determine homotypic doublets. The parameters pN (the number of artificial doublets) were set as 0.25, optimal pK (the neighborhood size) was determined by function “paramSweep_v3”, and nExp = round (0.0372*nrow (obj@meta.data)) (the number of expected real doublets) was adjusted by function “modelHomotypic”. The resulting cells annotated as “Singlets” in each library were kept for further analysis. Further quality control was subsequently performed as low‐quality cells were defined as expressing less than 700 genes. The libraries were then merged using Seurat. For normalization, the SCTransform was applied. 3000 most highly variable genes across samples were selected by the function “SelectIntegrationFeatures”. Finally, the function “IntegrateData” was employed to integrate multiple samples.

### Clustering and Cell Type Annotation for snRNA‐Seq Data

There were 2000 highly variable genes identified by “FindVariableFeatures”. Dimensionality reduction analysis was performed using “RunPCA” with default parameters, following “RunUMAP” and “RunTSNE”. The cell clusters were identified using function “FindNeighbors” with parameters dims = 1:33, and function “FindClusters” with a resolution of 0.5. Seurat dataset assay “RNA” was also normalized and scaled by function “NormalizeData” and “ScaleData”. To define genes enriched in each cluster, the “FindAllMarkers” function was used with parameters logfc.threshold = 0.05, min.pct = 0.1, min.diff.pct = 0.05. Wilcoxon rank‐sum test (*p* adj < 0.01) was used to define differentially expressed genes between each cluster and all other cells. The resultant genes were then used for GO enrichment analysis. Expression of marker genes published in single‐cell datasets^[^ ^15^ ^]^ (https://biobigdata.nju.edu.cn/scplantdb/home) and previous research^[^ ^16^, ^58^ ^]^ were visualized by function “Dotplot”. Cell type of each cluster was manually annotated according to the profile of marker genes, AddModuleScore function in Seurat, and the GO enrichment results. To define gene expression levels across specific cell populations, function “AvrageExpression” was employed using Seurat dataset assay “RNA”. Gene regulation across different cell populations was identified by function “FindMarker” using setting of only.pos = FALSE, test.use = “wilcox”. DEGs were then manually filtered as *p* adj < 0.01, |average_log_2_FC| > 1, and different pct.1, pct.2 conditions. The enrichment analysis of Kyoto Encyclopedia of Genes and Genomes (KEGG) pathways was performed using clusterProfiler (v4.10.1)^[^ ^59^ ^]^ with DEGs (|average_log_2_FC| > 1.0, *p* adj < 0.01, pct.1 > 0.1 for up‐regulated genes, and pct.2 > 0.1 for down‐regulated genes). Top 20 enriched pathways (based on adjusted *p*‐value) were displayed in plots. The pathways with adjusted *p*‐values greater than 0.05 were marked.

### Trajectory Inference and Pseudotime Analysis

To construct a trajectory, cells annotated with the same cell type were divided to mock group and *M. oryzae* group, which were fed into a Monocle2 pipeline, and the strategy of ordering single cells in pseudotime was applied.^[^ ^60^ ^]^ To avoid the impact of photoperiod, 12 h cells were ignored. The UMI count matrix was used to construct the dataset by the “newCellDataSet” function. The resultant dataset was processed to identify genes that were differentially expressed. The top 2000 significantly altered genes (according to the *q*‐values<0.01) from all *M. oryzae* datasets were selected to construct infection‐related trajectories. To minimize the impact of cell developmental effects on the trajectory construction, first, the top 500 significantly altered genes (according to the *q*‐values) from each mock dataset were selected, and overlapped genes (286 genes for epidermal cells, 236 genes for procambium cells) between 2000 treatment genes and 500 mock genes were removed. The resultant 1714 and 1764 genes were used to construct the infection‐related trajectory curves of epidermal cells and procambium cells, respectively. To build the trajectory and annotate each cell with a pseudotime, the “reduceDimension” function was applied with “DDRTree” method, followed by the function “orderCells”. Cells were then reordered by function “orderCells” with suitable “root_state” parameter to define the mock 0 h cells as the root of the trajectory. The top 2000 significantly altered genes (according to the *q*‐values, *q*‐value < 0.01) from infection‐related trajectories were clustered into 3 populations through hierarchical clustering. GO enrichment analysis was performed using clusterProfiler package^[^ ^59^ ^]^ with cut‐offs of *p* adj < 0.01.

### Bulk RNA‐Seq Data Analysis

To determine transcriptome changes in rice leaf during the infection by *M. oryzae*, raw RNA‐seq datasets PRJNA661210 were downloaded from a previous study.^[^ ^18^ ^]^ The raw reads were processed for quality checking and adaptor removal by fastp (https://github.com/OpenGene/fastp). Trimmed reads were aligned against the *O. sativa* genome (release 7 of the MSU) using Hisat2, followed by featureCounts^[^ ^61^ ^]^ to generate a count matrix. Differentially expressed genes were detected by DESeq2.^[^ ^62^ ^]^ DEGs were defined by a cut‐off of |log_2_FC| > 1, *p* adj < 0.01.

### Spatial Transcriptomics Sequencing

Spatial transcriptomics RNA sequencing was performed by BMKMANU S1000 NRA‐Seq. Due to the chip size limitation (0.68 cm × 0.68 cm), rice leaf fragments (2 cm) were rolled up to fit the space, with two biological replicates. All samples were embedded in OCT (SAKURA, 4583), and were cryosectioned at −20 °C. The BMKMANU S1000 RNA‐seq was performed with the BMKMANU S1000 Gene Expression kit (ST03002) and BMKMANU S1000 Tissue Optimization Kit (BMKMANU, ST03003), respectively. The optimized tissues were sequenced by Illumina NovaSeq 6000 with a sequencing depth of at least 50 000 reads per spot (100 µm) and 150 bp (PE150) paired‐end reads (performed by Biomarker Technologies Corporation, Beijing, China). The raw data from Illumina were mapped to the *O. sativa* subsp. Japonica cv. Nipponbare reference genome by BSTMatrix (v1.1), using default parameters. The spot image was loaded into the BSTViewer and corresponding level 6 (42 µm, 91 spots) matrix was used for downstream analysis.

### ALBST Construction and Split of Spatial Dataset

LOONG program was developed by C++, and was installed on Ubuntu 20.04. Command “draw_a_loong” was run to construct ALBST (Auxiliary line‐based spatial trajectory). The respective spots selected by BSTViewer were extracted from Seurat dataset first. Data from two layers of the leaf tissue was removed. The tissue image and the coordinates of spots were then input to LOONG. Spots were shown with the image background, and an auxiliary line was drawn along the curly leaf segments. For each spot, ALBST value and Width value were provided, which represent the relative position along and perpendicular to the auxiliary line, respectively. We first identified the nearest auxiliary line dot for each spot. Second, Euclidean distance between each spot and each nearest dot (Width value) was calculated. “θ” was used to define the direction of spot. The spot located on the left side of ALBST (θ < 180°, Width>0) or located on the right side (θ > 180°, Width<0). Finally, the curve length from the nearest dot to the first dot was defined as ALBST value. ALBST was scaled to 2 cm, defined into “−1” to “1”, to fit the actual length of leaf fragments, and width was also equally scaled. Here, “0” was defined as inoculation site. Spots of each leaf segment were then divided evenly into 40 fragments from “−1” to “1” of ALBST values. Then, mean 942 genes were detected for each spot for the quality control (QC), and at least 10 spots were qualified for each fragment (500 µm), which gives mean 8000 genes for each fragment. The stRNA‐seq data was analyzed by the number of detectable genes versus the number of total detectable genes in each leaf fragment (Expression ratio). Spatial datasets were first normalized and scaled by function “NormalizeData” and “ScaleData”. The function “AvrageExpression” was then employed to define genes expression levels across ALBST clusters. Numbers of detectable genes (mean expression > 20) of different gene sets were counted in each ALBST cluster. The ratio of detectable genes was then fitted by the function “gam”. Spatial dataset of each leaf sample was fed into Monocle2, and genes of mean expression > 0.1 were selected to construct a normal trajectory and annotate each spot with a pseudotime. Then, the pseudotime values were directly replaced with ALBST values. The function “plot_pseudotime_heatmap” was employed to visualize the spatial expression pattern of ion transport‐related genes along ALBST.

### Measurement of Momilactone A by HPLC‐MS/MS

Momilactone A extraction and analysis were performed by modification of the method described previously.^[^ ^29^ ^]^ The infected rice leaves were harvested and the vascular and mesophyll tissues were separated according to the method described by Endo et al.^[^ ^63^ ^]^ One hundred milligrams of 2‐week‐old seedlings were cut to 0.5‐mm fragments and hydrolyzed in cellulase R‐10 and macerozyme R‐10 enzyme solution for 30 min. Then the solution was incubated at 80 rpm, 28 °C for 4 h. The resulted tissues were gently grounded and filtered by 25‐µm filter cloth to separate vascular tissues (Vt) and other tissues (Ot). Vt, which accidentally mixed into Ot, was removed by needle under microscope. The mixture was transferred into centrifuge tube and centrifuged at 300 g min^−1^ for 5 min. The pellets were Ot. All samples were lyophilized and the resulting residue was homogenized in liquid nitrogen. The tissues were dissolved in 1 mL of methanol: H_2_O (90:10, v/v, add 0.1% formic) for 12 h at 4 °C. After centrifugation, the supernatant was collected. The solvent was evaporated, and the residue was dissolved in 100 µL HPLC grade methanol. Two microliters of samples were subjected to HPLC‐MS/MS analysis on an Agilent 1260 HPLC coupled to a QTRAP 4500 mass spectrometer equipped with an electrospray interface (AB SCIEX, CA, US). Samples were eluted with gradient mobile phase [methanol: H_2_O, 20:80 (v/v)] for 10 min, 95:5 (v/v) in 10–15 min, and 20:80 (v/v) in 15–20 min at a flow rate of 0.4 mL min^−1^ on the Phenomenex Synergi Hydro‐RP Column (150 mm × 2 mm, 4 µm). Multiple reaction monitoring of ion pairs in positive mode was used for analysis of momilactone A (315.0>271.0). The content was expressed as ng g^−1^ of dry weight. All assays were performed with three independent biological replicates.

### Paraffin Sectioning and SEM Observation

For paraffin sectioning, the samples were processed according to the previously described method.^[^ ^64^ ^]^ Rice leaf fragments (0.5 cm) were fixed in solution FAA (Formaldehyde‐acetic acid‐ethanol Fixative) for 48 h at 4 °C. The samples were dehydrated sequentially in 50%, 70%, 85%, 95%, and 100% ethyl alcohol for 30 min of each solution. Next, the samples were hyalinized under sequential ethyl alcohol and xylene mixtures (ethyl alcohol: xylene = 3:1, 1:1, 1:3, v/v) for 40 min of each solution. Then, the leaf fragments were embedded in paraffin and were sliced into 8‐µm thick sections. The tissue was observed under a light microscope (Olympus, SZ680). For the scanning electron microscope (SEM) observation, the fixed tissues were dried using a critical point dryer (Tousimis Samdri‐PVT‐3D, USA). The samples were coated with platinum by the ion sputter (HITACHI E‐1010, Japan) and observed under the scanning electron microscope (SEM, ZEISS Crossbeam 550, Germany).

### RT‐qPCR and qPCR Assays

Total RNA isolation and real‐time PCR assays were performed according to the previously described method.^[^ ^65^ ^]^ Briefly, rice leaves that were infected by blast fungi were used to extract mRNA with TRIzol Universal reagent (Tiangen). Then, 4×gDNA wiper Mix was used to remove DNA. 5×HiScript II qRT SuperMix II (Vazyme Biotech) was used to transcribe mRNA to cDNA. The RT‐qPCR assays were performed with 2×Taq Pro Universal SYBR (Vazyme Biotech) on QuantStudio1 (Thermo, USA). *OsActin1* was used as the internal control.

After inoculated with *M. oryzae*, infected rice leaves were sampled for fungal biomass measurement. For fungal biomass measurement in vein and other tissues, we separated vein tissues by tweezers under microscope. CTAB was used to extract total genomic DNA, and determined by qPCR against *MoPot2* gene of blast fungi and rice gene *OsUbi1*,^[^ ^66^ ^]^ respectively. The primers were listed in Table S11 (Supporting Information).

### Fungal Infection Assays and Confocal Microscope Observation

Rice sheath inoculation assays were performed as previously described.^[^ ^29^ ^]^ Ten rice sheaths were injected with conidial suspensions at a concentration of 5×10^5^ spores mL^−1^ for each treatment. The Guy11‐mCherry strain was used for inoculation. The inoculated sheath was observed with a confocal microscope (Zeiss, LSM‐800) at 12, 24, 48, and 72 hpi. Same concentration of the Guy11‐mCherry conidial suspensions was spray‐inoculated on *35S:PIP2‐GFP* transgenic plant leaves to observe the infection process in rice tissues.

### Statistical Analysis

All data are shown as mean ± standard deviation (SD) from no less than three biological repeats. Two‐tailed Student's *t*‐test with GraphPad Prism v8.0 was used for comparing means between two samples (*, **, and *** represent at *p*≤ 0.05, 0.01, and 0.001 levels, respectively). N = 3 for analysis of qRT‐PCR data; *n* = 5 for analysis of relative fungal biomass; *n* = 81 for statistic assays of rice blast lesions in field. One‐way ANOVA (Analysis of variance) with GraphPad Prism v8.0 was used for examining the significance of the difference among different groups (different letters indicate significant differences, *p* < 0.05).

## Conflict of Interest

The authors declare no conflict of interest.

## Author Contributions

W.W., X.Z., Y.Z., and Z.Z. contributed equaly to this work. J.L. and N.X. conceived and designed the experiments; W.W., X.Z., Y.Z., J.K., C.Y., W.C., Q.Z., Q.H., Y.L., and Y.Z. performed most of the experiments and data analysis; W. Q. and N.Y. provided valuable advices and discussions; W.W., N.X., and J.L. wrote the article.

## Supporting information



Supporting Information

Supplemental Video 1

Supplemental Video 2

Supplemental Video 3

Supplemental Table 1

Supplemental Table 2

Supplemental Table 3

Supplemental Table 4

Supplemental Table 5

Supplemental Table 6

Supplemental Table 7

Supplemental Table 8

Supplemental Table 9

Supplemental Table 10

Supplemental Table 11

## Data Availability

The raw snRNA and stRNA sequencing data generated in this study can be obtained from the National Center for Biotechnology Information PRJNA1121079 and PRJNA1150774.
