# Supplementary material for: Single‐Cell and Spatial Transcriptomics Reveals a Stereoscopic Response of Rice Leaf Cells to Magnaporthe oryzae Infection
Source: Adv Sci (Weinh). 2025 Mar 24;12(19):2416846. doi: 10.1002/advs.202416846 (PMC12097076; doi:10.1002/advs.202416846)
Supplement: Supplementary file 1 — Supporting Information [file ADVS-12-2416846-s013.pdf]

ADVANCED SCIENCE
Open Access

Supporting Information

for *Adv. Sci.*, DOI 10.1002/advs.202416846

Single-Cell and Spatial Transcriptomics Reveals a Stereoscopic Response of Rice Leaf Cells to *Magnaporthe oryzae* Infection

Wei Wang, Xianyu Zhang, Yong Zhang, Zhe Zhang, Chang Yang, Wen Cao, Yuqin Liang, Qinzheng Zhou, Qian Hu, Yimai Zhang, Yu Wang, Yingying Xing, Wenfeng Qian, Nan Yao, Ning Xu and Jun Liu**

Supplementary Fig. 1

a

Sample	Number of replicates	Mean of UMI	Number of cells	Mean gene per cell
Mock 0 h	2	1,974	13,343	1,106
Mock 12 h	2	3,991	21,656	1,532
Mock 24 h	2	2,039	26,046	1,079
Mock 48 h	2	2,267	35,324	1,210
M.o 0 h	2	3,114	28,736	1,489
M.o 12 h	2	3,096	25,914	1,410
M.o 24 h	2	2,464	27,328	1,175
M.o 48 h	2	2,851	58,361	1,458

b

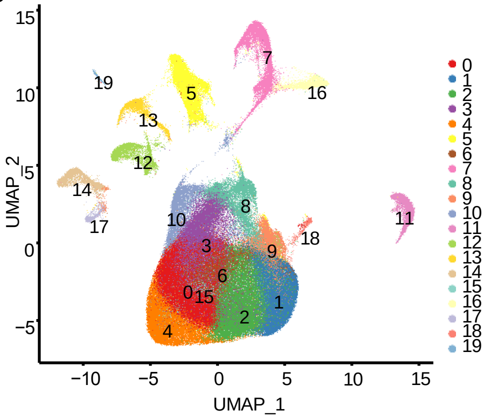

c

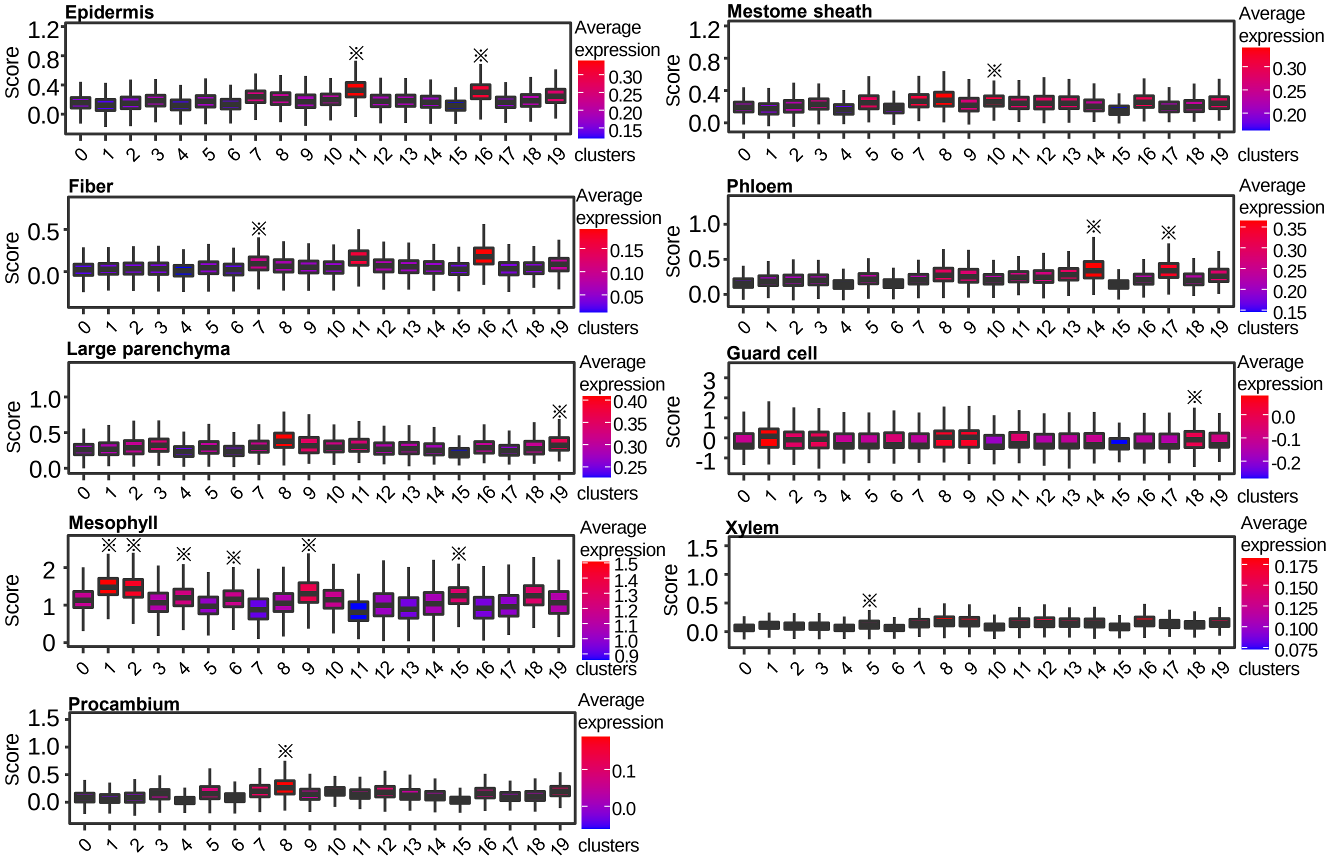

Supplementary Fig. 1 Cell types annotation of rice leaves during *M. oryzae* infection

(a) The snRNA-seq information of the samples. Two-week-old rice leaves were inoculated with *M. oryzae* spores at a concentration of $5{\times}10^5$ spores/mL. The samples were taken at 0, 12, 24, and 48 hpi (hours post inoculation) for snRNA-seq. Mock is the solvent control (0.02% tween-20). Two biological samples were used for snRNA-seq and the data analysis. UMI, unique molecular identifier.

(b) UMAP visualization of rice leaf single cell atlas. The cells were divided into 20 clusters according to Graph-based unsupervised clustering. Each dot represents the transcriptional signature of a single cell. The dot color indicates the clusters.

(c) Boxplot shows the expression features in 20 clusters using AddModuleScore function in Seurat. “※” indicates identified cell types in 20 cluster.

Supplementary Fig. 2

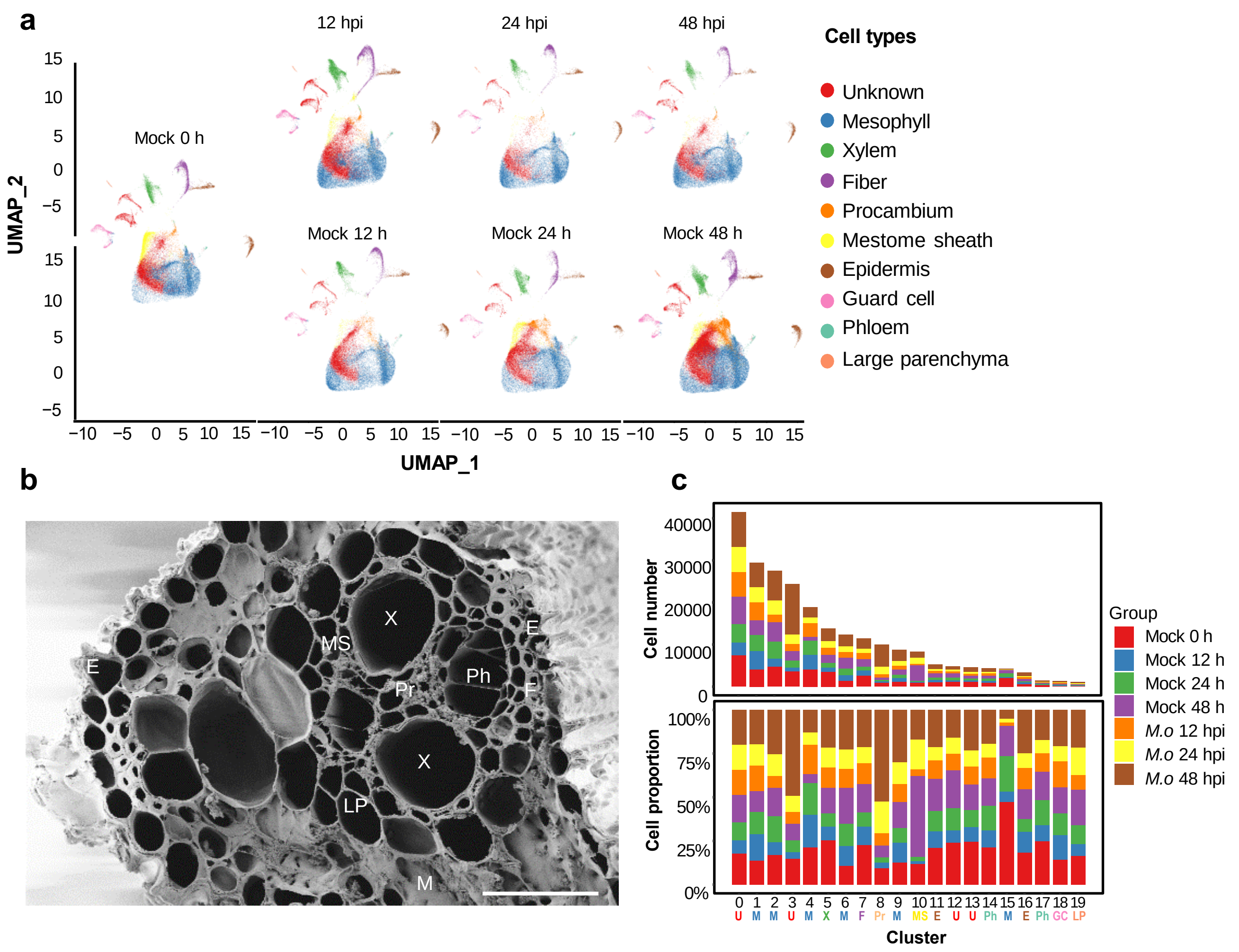

Supplementary Fig. 2 Cell transcriptome atlas of rice leaves during *M. oryzae* infection

(a) Transcriptome changes in response to *M. oryzae* infection captured by snRNA-seq. The separated single-cell atlas was generated from mock-treated and *M. oryzae*-infected leaves at the indicated time points. Mock is the solvent control.

(b) The scanning electron microscope image of the cross dissection of rice leaf vein. Two-week-old rice leaf cross dissection of vein was used and the image was taken with SEM. Bar = 10 µm.

(c) Proportions of cells from 0, 12, 24, and 48 hpi samples and their respective control samples in each cluster. Upper panel: the total cell numbers of each cluster; Lower panel: the proportions were normalized against the total number of cells in each cluster. M, Mesophyll; X, Xylem; F, Fiber; Pr, Procambium; MS, Mestome sheath; E, Epidermis; GC, Guard cell; Ph, Phloem; LP, Large parenchyma; U, Unknown.

Supplementary Fig. 3

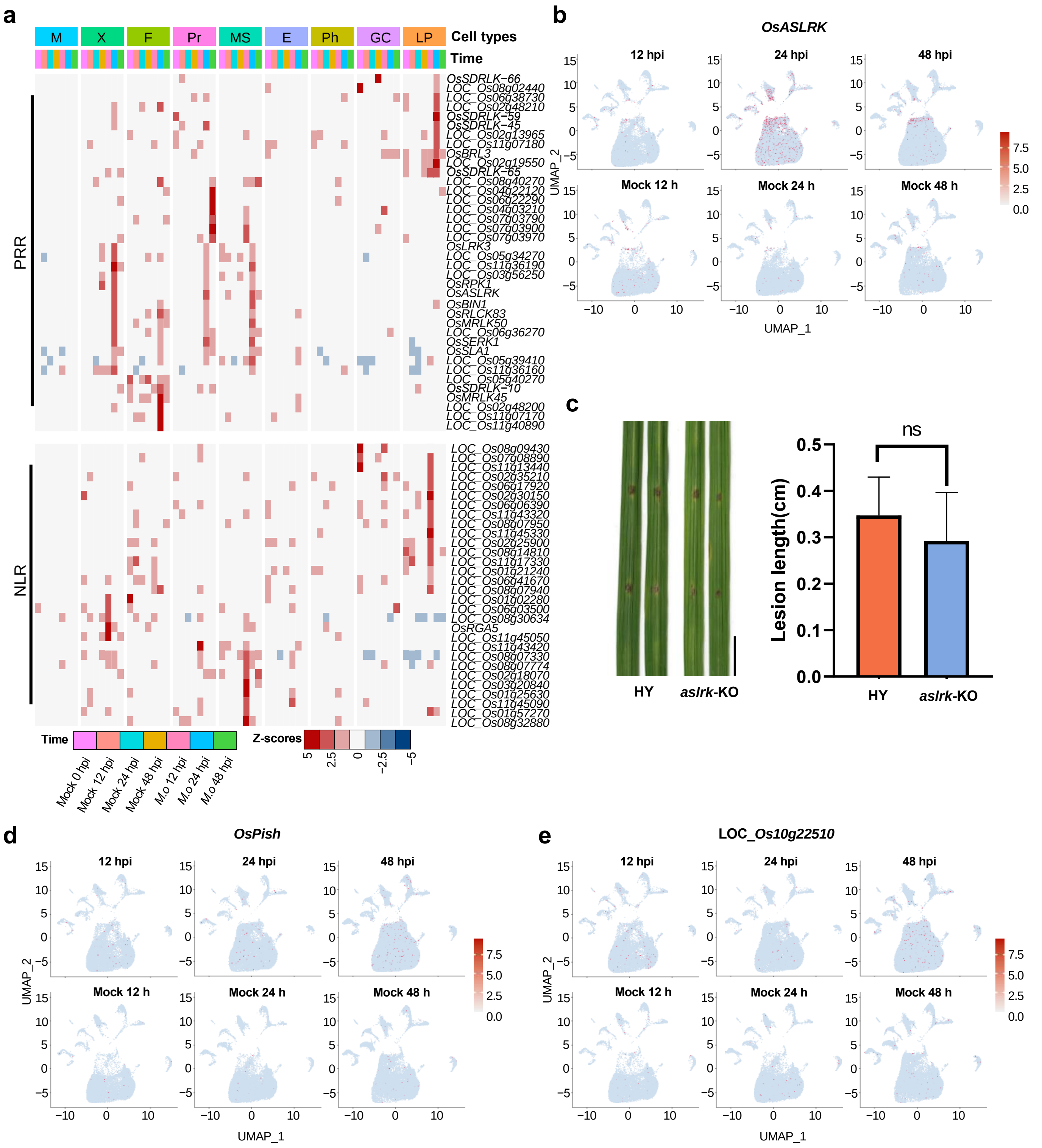

Supplementary Fig. 3 Cell-type-specific expression of immune genes

(a) Cell-type-specific expression of PRRs and NLRs during *M. oryzae* infection. Heatmaps show mean expression levels of known or predicted PRR and NLR-encoding genes from rice in each annotated cell type at indicated infection time points. The mean gene expression levels are indicated by colored Z-score. M, Mesophyll; X, Xylem; F, Fiber; Pr, Procambium; MS, Mestome sheath; E, Epidermis; GC, Guard cell; Ph, Phloem; LP, Large parenchyma.

(b) UMAP visualization of expression patterns of PRR gene *OsASLRK* at 12, 24, and 48 hpi. Each dot represents a single cell.

(c) Disease symptom and lesion length of *aslrk*-KO mutant after punch inoculation with *M. oryzae* spores. The control is rice variety HY (HwaYong background). The conidial suspensions at a concentration of 5×10^5 spores/mL were spotted onto the leaf surface of one-month-old plants. After 5 days of inoculation, the disease symptom was imaged. Bar = 1 cm. ns, no significant (Student's *t*-test, n= 4 lesions).

(d) and (e) UMAP visualization of expression patterns of NLR gene *OsPish* (d) and *LOC_Os10g22510* (e) at 12, 24, and 48 hpi. The UMAP was constructed using snRNA-seq data. The red color indicates the gene expression levels in cells.

Supplementary Fig. 4

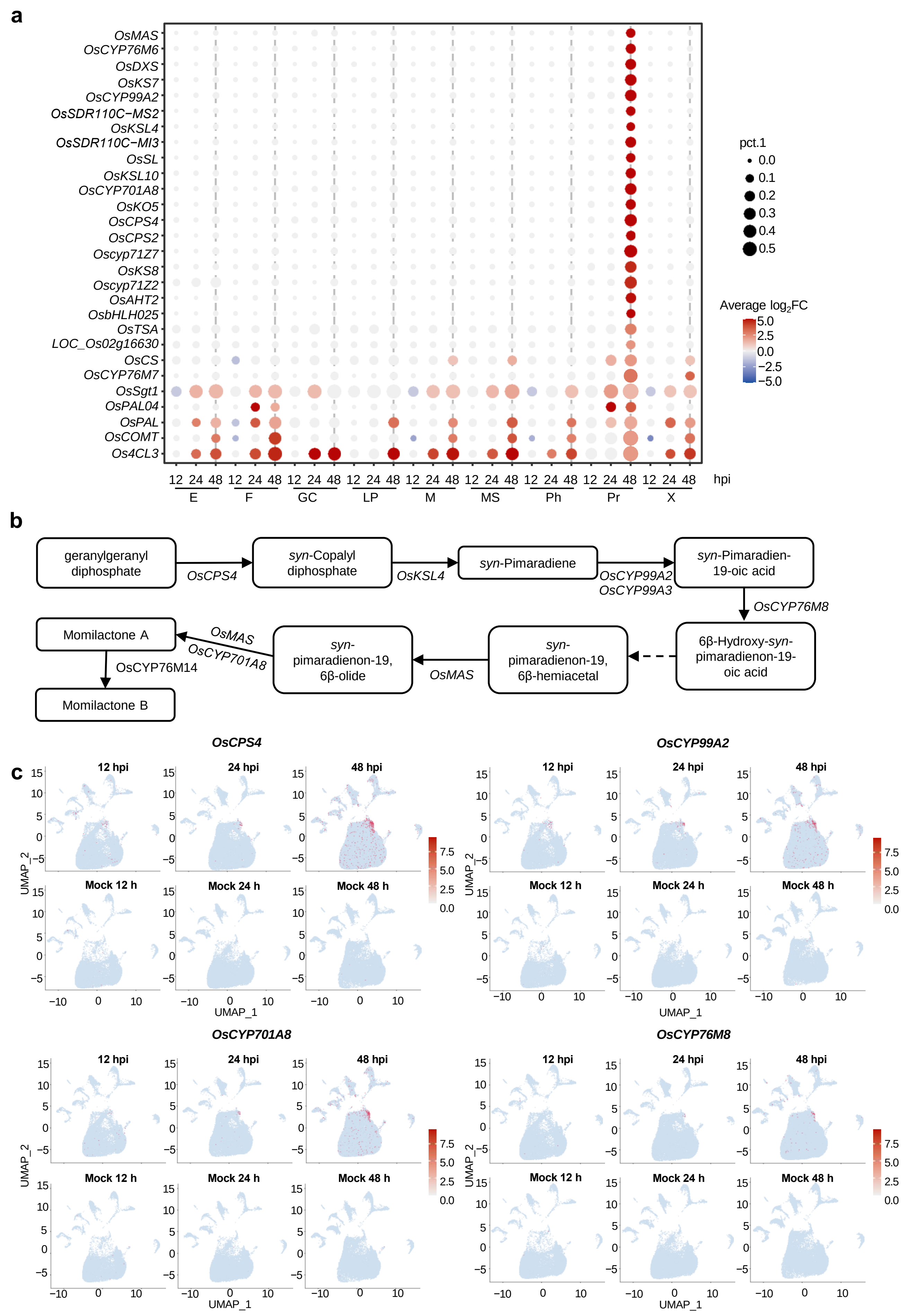

Supplementary Fig. 4 Expression of metabolite biosynthesis-related genes in rice leaf during blast infection

(a) Cell-specific expression of terpene biosynthesis-related genes. The expression of terpene biosynthesis-related DEGs was shown by dotplot landscape from the snRNA-seq data. The parameters are set as |average $\log_2$FC|>1.0, *p* adj<0.01, pct.1>0.1 for up-regulated genes and pct.2>0.1 for down-regulated genes. M, Mesophyll; X, Xylem; F, Fiber; Pr, Procambium; MS, Mestome sheath; E, Epidermis; GC, Guard cell; Ph, Phloem; LP, Large parenchyma. pct.1 and pct.2 meant the percentage of cells where the gene is detected in the treatment and mock groups, respectively.

(b) Momilactone biosynthesis pathway in rice. The key momilactone biosynthesis genes and their substrates and products were shown (Ref Kato-Noguchi, 2023).

(c) UMAP visualization of expression patterns of momilactone A biosynthesis genes at 12, 24, and 48 hpi in transcriptome atlas. The red color indicate the gene expression levels in cells.

Supplementary Fig. 5

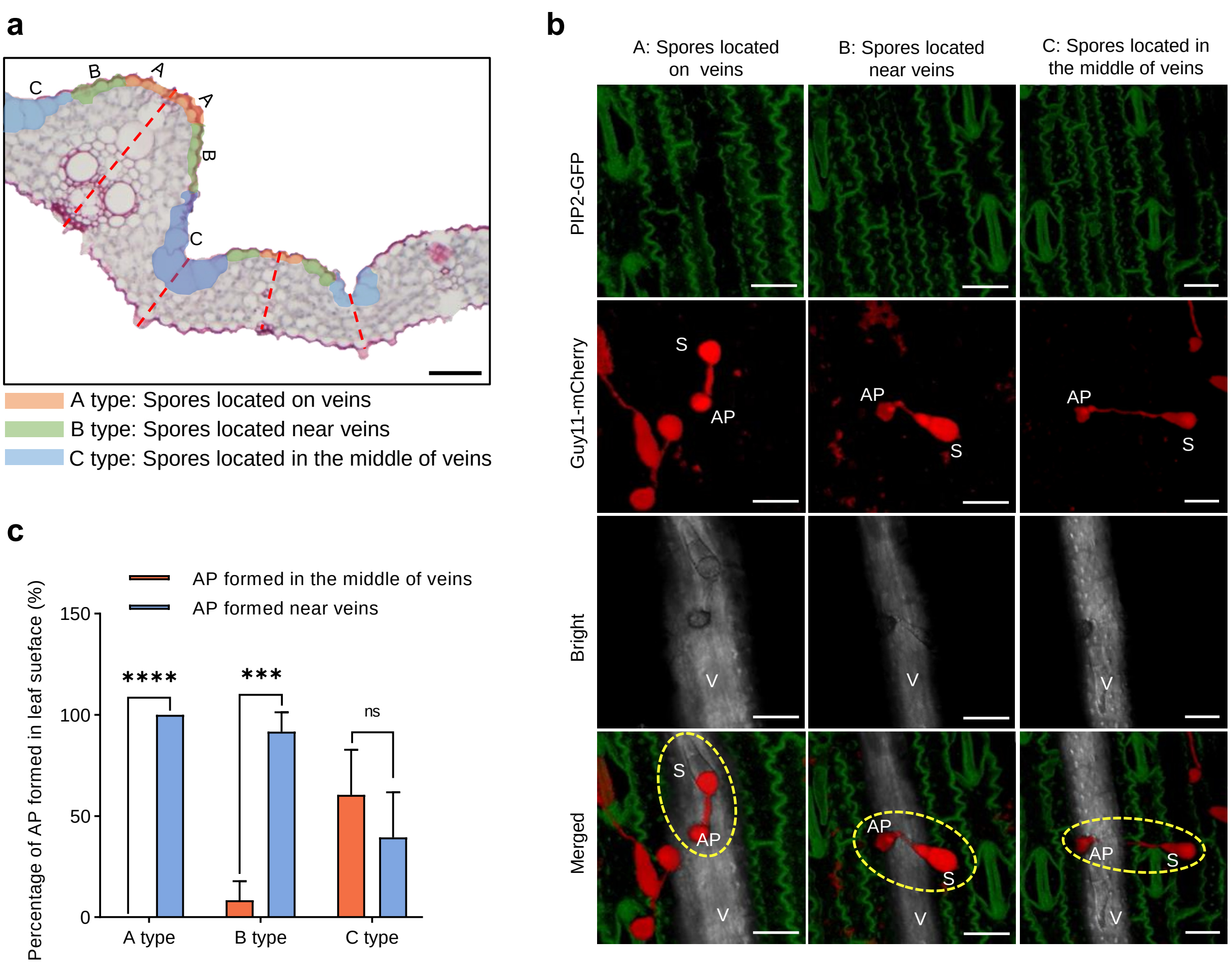

Supplementary Fig. 5 ***M. oryzae*** **prefers to form appressorium in rice leaf vein**

(a) The model of the divided regions of rice leaf surface. The orange areas indicate the location of spores from *M. oryzae* on vein regions (A type); The green areas indicate the location of spores from *M. oryzae* near vein regions (B type). The blue areas indicate the location of spores from *M. oryzae* in the middle of vein regions (C type); Scale bar, 20 μm.

(b) Representative confocal micrographs show that *M. oryzae* targets rice leaf veins at appressorium formation stage. The mCherry-labeled *M. oryzae* strain Guy11 primarily targeted rice leaf veins at 24 hours post inoculation (hpi). The rice cell plasma membrane was labeled with PIP2-GFP to visualize cell profile. The rice leaves were spray-inoculated with *M. oryzae* spores at a concentration of 5×10^5 spores/mL. V, vein; S, spore; AP, appressorium. The yellow dashed circle highlights germination of *M. oryzae* spores; Scale bars, 10 μm.

(c) Statistic analysis of appressorium formation of *M. oryzae* in rice leaf surface. Three types were indicated as spores located on vein regions (A type), near vein regions (B type), and in the middle of vein regions (C type). Percentages were presented the appressorium formation in the middle of veins and nearby veins in rice leaf surface. AP, appressorium. "***", significant, p=0.0006; "****", significant, p<0.0001; ns, no significant (Student's t-test, total n= 100).

Supplementary Fig. 6

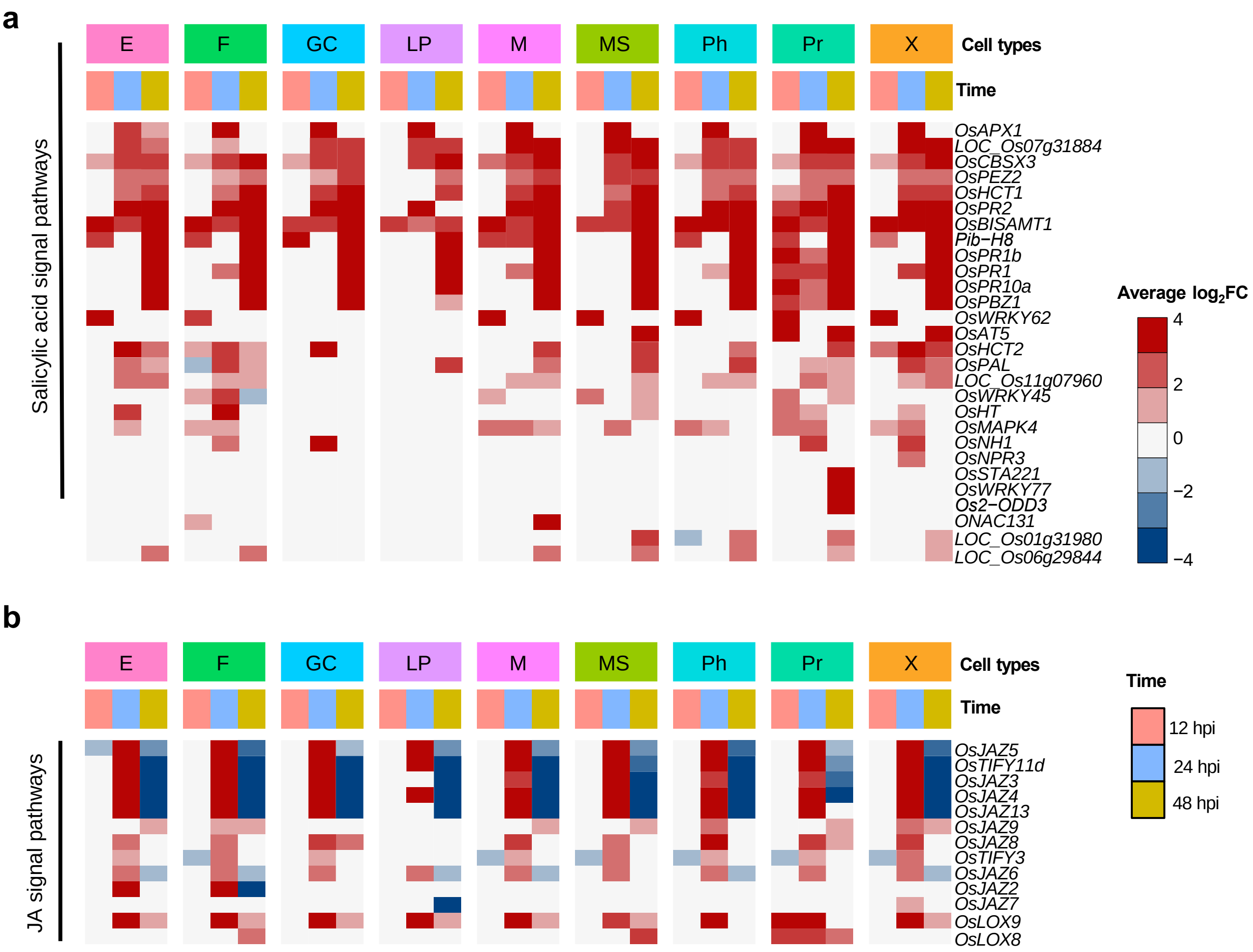

Supplementary Fig. 6 Cell-type-specific expression of salicylic acid and jasmonic acid signaling-related genes

(a) Cell-type-specific expression of salicylic acid (SA) signaling pathway-related genes during *M. oryzae* infection. Heatmaps show the relative expression of salicylic acid signaling pathways-related DEGs in each annotated cell population of rice leaves. M, Mesophyll; X, Xylem; F, Fiber; Pr, Procambium; MS, Mestome sheath; E, Epidermis; GC, Guard cell; Ph, Phloem; LP, Large parenchyma.

(b) Cell-type-specific expression of jasmonic acid (JA) signaling pathway-related genes during *M. oryzae* infection. Heatmaps show the relative expression of JAZs-encoding genes in each annotated cell population of rice leaves. M, Mesophyll; X, Xylem; F, Fiber; Pr, Procambium; MS, Mestome sheath; E, Epidermis; GC, Guard cell; Ph, Phloem; LP, Large parenchyma.

The color intensity represents average Log_2FC (|average log_2FC|>1.0, *p* adj <0.01, pct.1>0.1 for up-regulated genes and pct.2>0.1 for down-regulated genes). M, Mesophyll; X, Xylem; F, Fiber; Pr, Procambium; MS, Mestome sheath; E, Epidermis; GC, Guard cell; Ph, Phloem; LP, Large parenchyma.

Supplementary Fig. 7

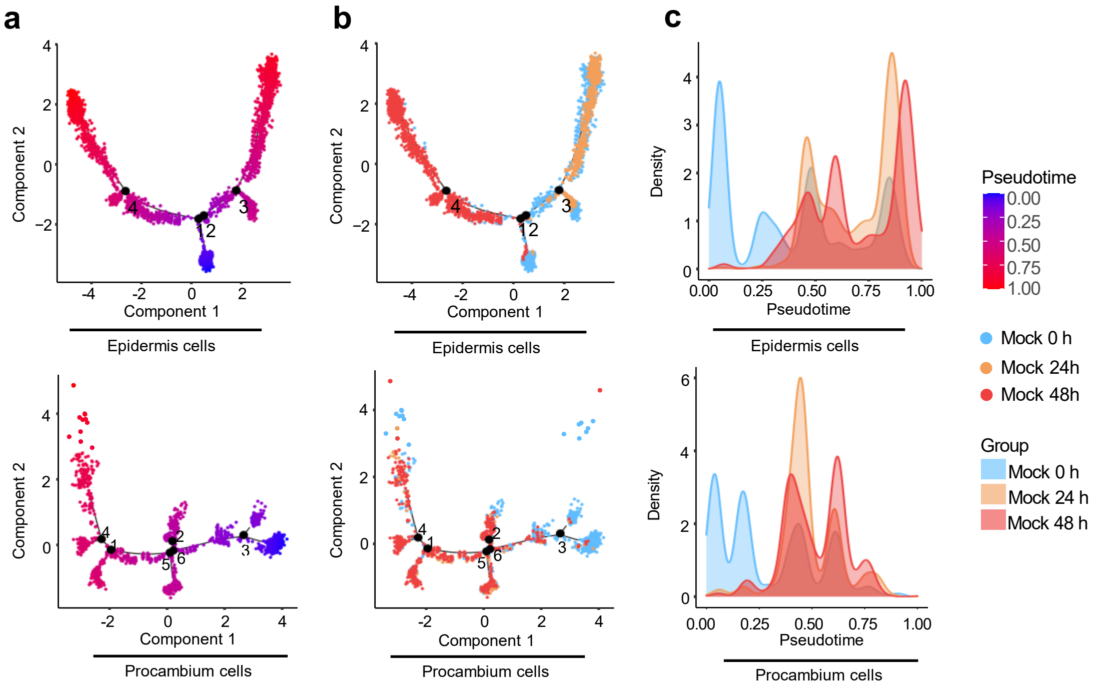

Supplementary Fig. 7 Pseudotime assays of rice leaf cells by mock treatments in epidermis cells and procambium cells

(a) The trajectory curve of epidermal and procambium cells from the mock-treated rice leaf cells. The color of a dot indicates its pseudotime value.

(b) The trajectory curve of epidermal and procambium cells from the mock-treated cells at indicated time points. The color of a dot indicates the sampling time after treatment.

(c) The curve uniformity disagrees with pseudotime and treatment time in mock samples. A shift of pseudotime was set from “0” to “1” in the cells. The color indicates the sample time points by mock treatment.

Each dot represents a single cell. Same set of the genes used for infected cells of trajectory curves were employed to construct the curves for mock-treated cells. Number “1” to “6” indicate the branching points.

Supplementary Fig. 8

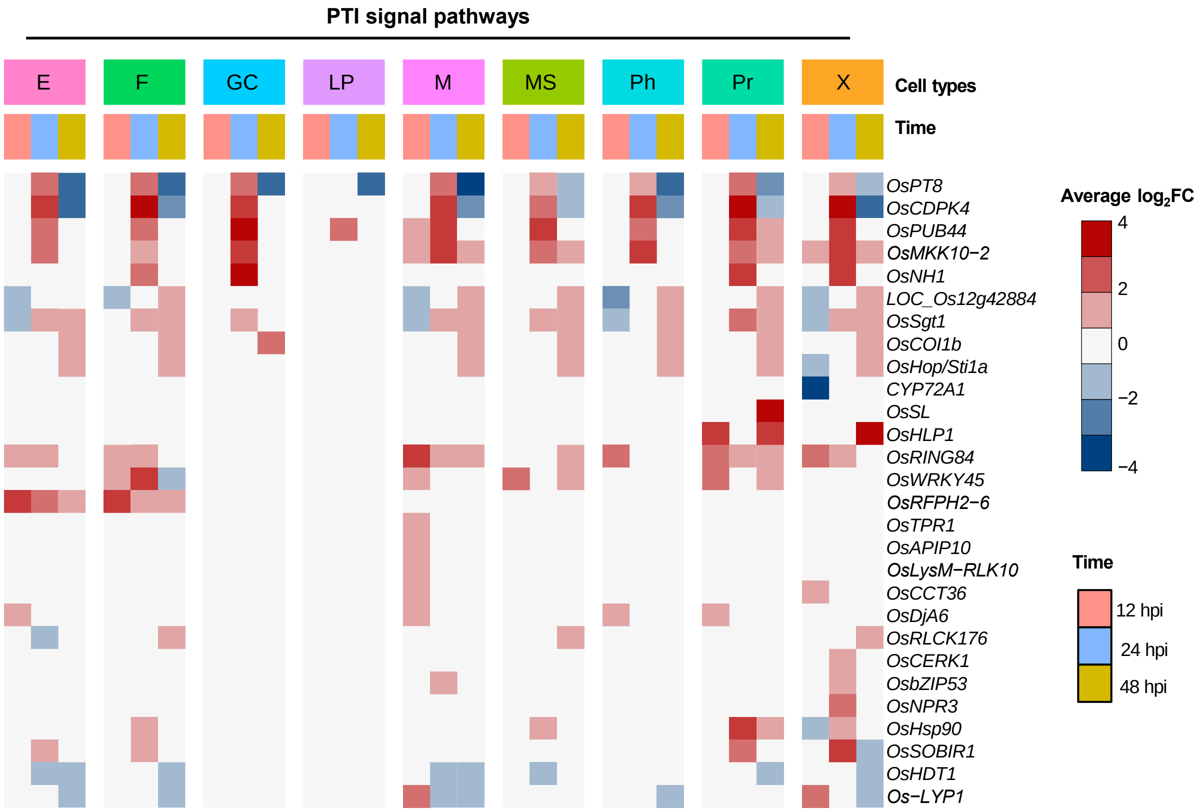

Supplementary Fig. 8 Cell-type-specific expression of PTI signaling-related genes

Cell-type-specific expression of PTI signaling pathway-related genes during *M. oryzae* infection. Heatmaps show the relative expression of PTI signaling pathways-related DEGs in each annotated cell population of rice leaves. The color intensity represents average Log_2FC (|average $\log_2\text{FC}$|>1.0, *p* adj <0.01, pct.1>0.1 for up-regulated genes and pct.2>0.1 for down-regulated genes). M, Mesophyll; X, Xylem; F, Fiber; Pr, Procambium; MS, Mestome sheath; E, Epidermis; GC, Guard cell; Ph, Phloem; LP, Large parenchyma.

Supplementary Fig. 9

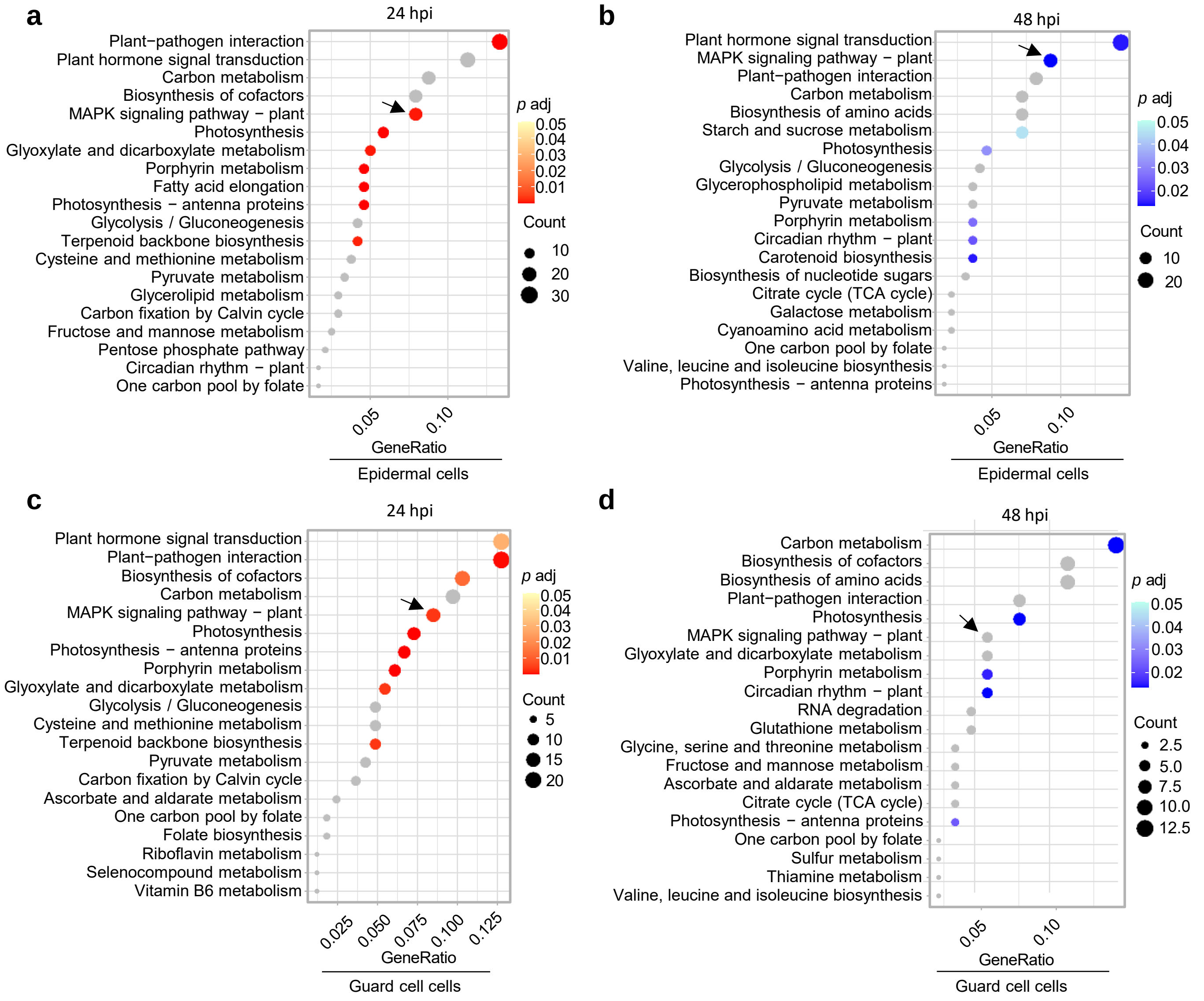

Supplementary Fig. 9 KEGG pathway analysis of DEGs in epidermis cells and guard cells of rice leaf under inoculated with *M. oryzae*

(a) to (d) KEGG pathway analysis of DEGs in epidermal cells and guard cells. The MAPK pathways were significantly enriched at 24 hpi in epidermal cells (a) and guard cells (c), and down regulation at 48 hpi in epidermal cells (b) and guard cells (d). Red bar indicates the upregulation of the DEGs and blue bar indicates the down regulation of the DEGs. *p* adj <0.01, pct.1>0.1 for up-regulated genes, and pct.2 >0.1 for down-regulated genes, |average $\log_2$FC|>1.0. MAPK pathways were indicated by the black arrowheads.

Supplementary Fig. 10

a

Sample	Number of replicates	Median UMI counts per spot(L6)	Mean UMI counts per spot(L6)	Median genes per spot(L6)	Mean genes per spot(L6)
Mock 0h	2	648	766	433	474
Mock 6h	2	1,404	1,678	949	1,059
Mock 12h	2	805	1,296	630	860
Mock 24h	2	1,902	2,108	1,064	1,121
M.o 6h	2	1,527	1,957	1,034	1,204
M.o 12h	2	619	1,073	507	738
M.o 24h	2	1,691	2,069	999	1,139

b

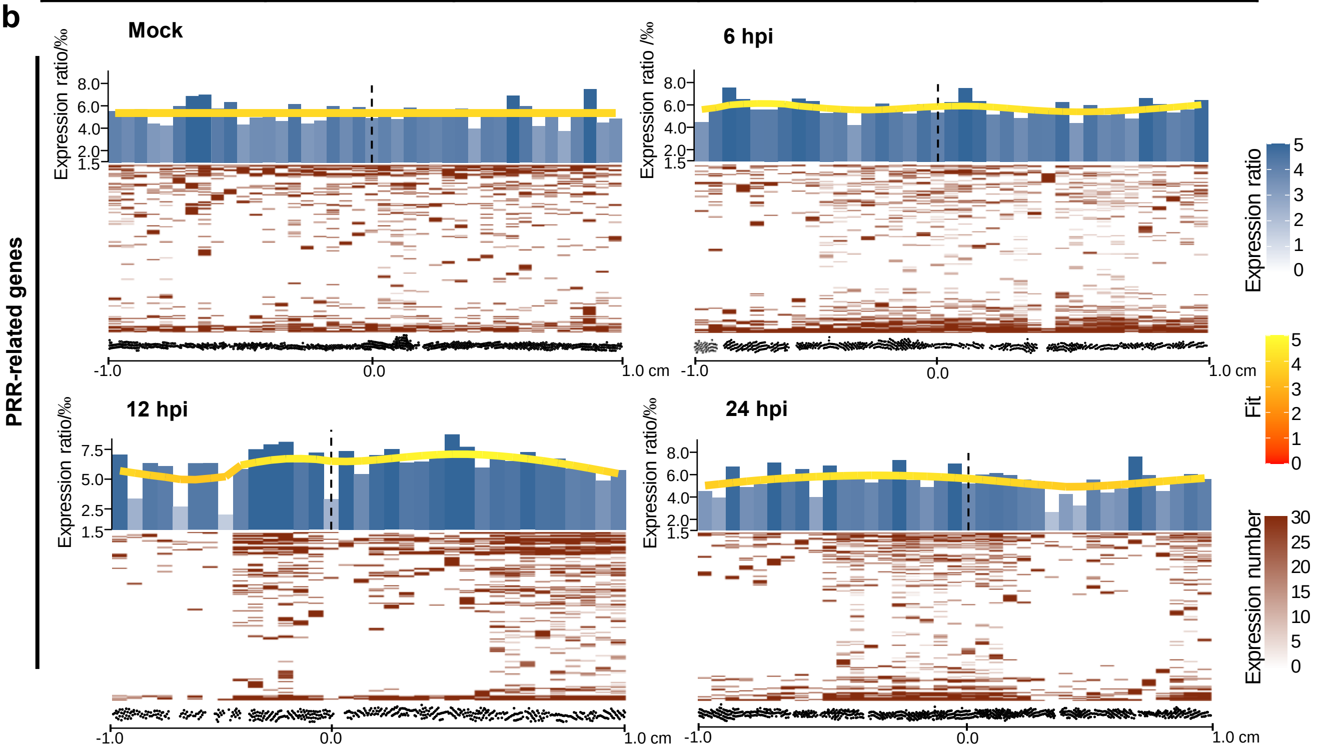

Supplementary Fig. 10 Spatial transcriptome revealed expression patterns of PRR-related genes in rice leaves during *M. oryzae* infection

(a) The stRNA-seq information of the samples. Two-week-old rice leaves were inoculated with *M. oryzae* spores at a concentration of 5×10^5 spores/mL. The samples were taken at 0, 6, 12 and 24 hpi (hours post inoculation) for stRNA-seq. Mock is the solvent control (0.02% tween-20). Two biological samples were used for stRNA-seq and the data analysis. UMI, unique molecular identifier.

(b) Expression ratio profile of PRR-related genes in *M. oryzae*-infected rice leaves (2.0 cm). The gene expression profiles were linearized and analyzed at 0, 6, 12, and 24 hpi. The color intensity indicates the gene expression ratio. The fit lines represents a fitted expression ratio value calculated by R function “mgcv::gam”. The inoculation site was located at “0 cm”. The “-1.0 cm” is the direction towards leaf base, and “1.0 cm” is to leaf tip. Expression ratio indicates the number of detectable PRR genes vs the number of total detectable genes in each leaf fragments. “Dotted line” indicates infection site.

Supplementary Fig. 11

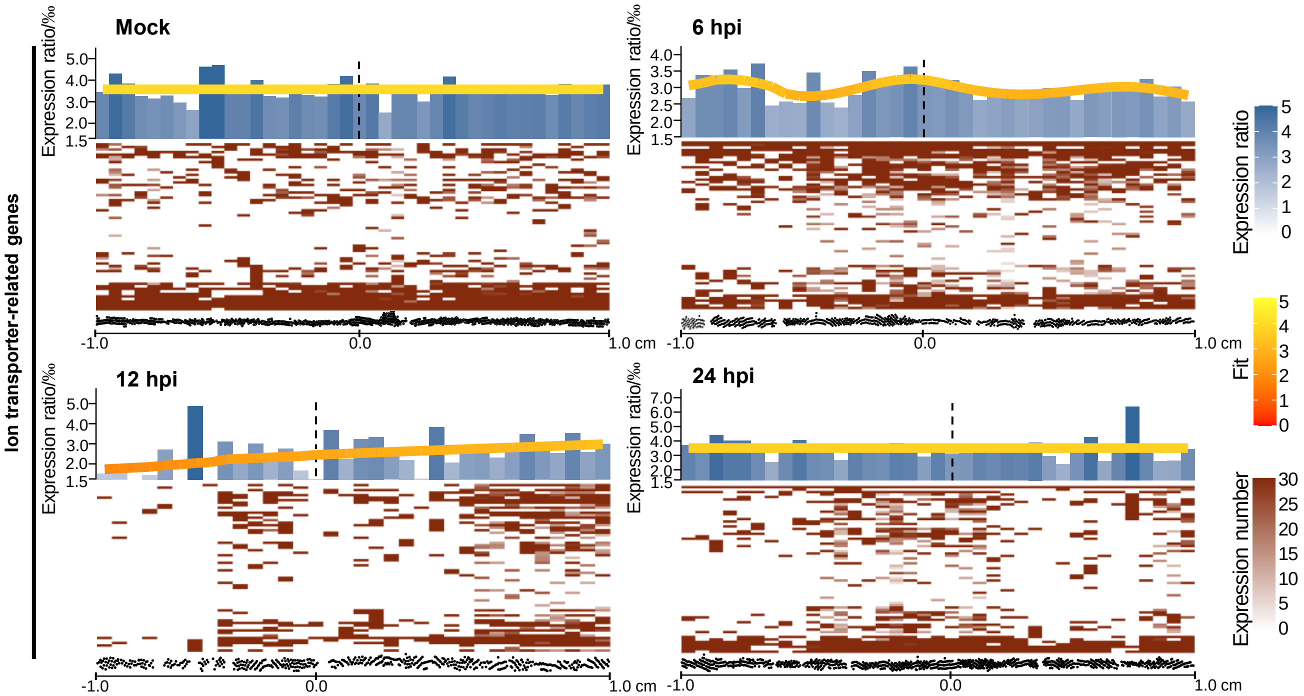

Supplementary Fig. 11 Spatial transcriptome revealed expression patterns of ion transport-related genes in rice leaves during *M. oryzae* infection

Expression ratio profile of ion transporter-related genes in *M. oryzae*-infected rice leaves (2.0 cm). The gene expression profiles were linearized and analyzed at 0, 6, 12 and 24 hpi. The color intensity indicates the gene expression ratio. The fit lines represents a fitted expression ratio value calculated by R function “mgcv::gam”. The inoculation site was located at “0 cm”. The “-1.0 cm” is the direction towards leaf base, “1.0 cm” is to leaf tip at 0, 6, 12 and 24 hpi. Expression ratio indicates the number of detectable ion transporters vs the number of total detectable genes in each leaf fragments. “Dotted line” indicates infection site.

Supplementary Video 1

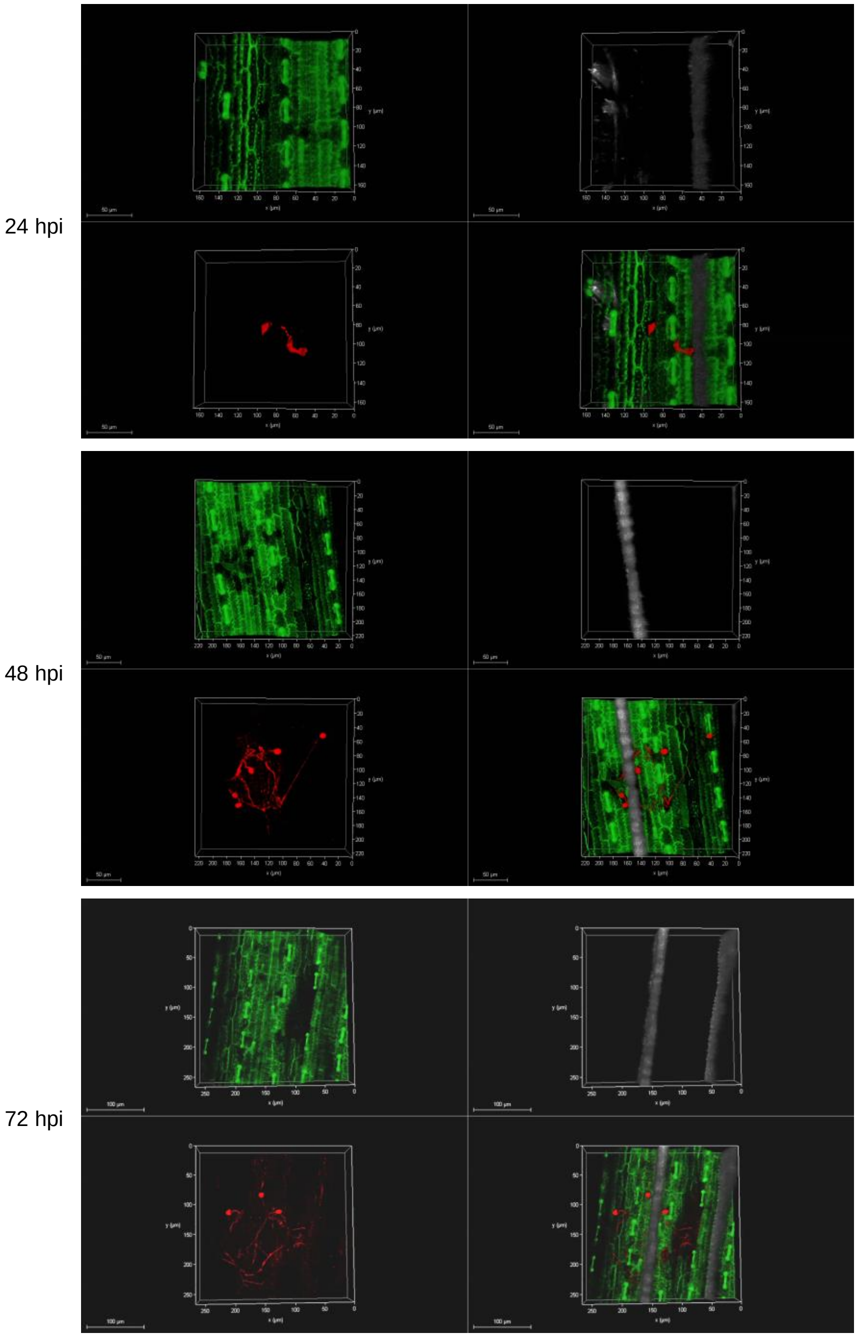

Supplementary videos: The 3-D images of *M. oryzae* infection process

The Guy11-mCherry was used to inoculate the leaf sheath at a concentration of 5×10^5 spores/mL. The rice plasma membrane was labeled with PIP2-GFP to show the cell profile.
